# A chickpea genetic variation map based on the sequencing of 3,366 genomes

**DOI:** 10.1038/s41586-021-04066-1

**Published:** 2021-11-10

**Authors:** Rajeev K. Varshney, Manish Roorkiwal, Shuai Sun, Prasad Bajaj, Annapurna Chitikineni, Mahendar Thudi, Narendra P. Singh, Xiao Du, Hari D. Upadhyaya, Aamir W. Khan, Yue Wang, Vanika Garg, Guangyi Fan, Wallace A. Cowling, José Crossa, Laurent Gentzbittel, Kai Peter Voss-Fels, Vinod Kumar Valluri, Pallavi Sinha, Vikas K. Singh, Cécile Ben, Abhishek Rathore, Ramu Punna, Muneendra K. Singh, Bunyamin Tar’an, Chellapilla Bharadwaj, Mohammad Yasin, Motisagar S. Pithia, Servejeet Singh, Khela Ram Soren, Himabindu Kudapa, Diego Jarquín, Philippe Cubry, Lee T. Hickey, Girish Prasad Dixit, Anne-Céline Thuillet, Aladdin Hamwieh, Shiv Kumar, Amit A. Deokar, Sushil K. Chaturvedi, Aleena Francis, Réka Howard, Debasis Chattopadhyay, David Edwards, Eric Lyons, Yves Vigouroux, Ben J. Hayes, Eric von Wettberg, Swapan K. Datta, Huanming Yang, Henry T. Nguyen, Jian Wang, Kadambot H. M. Siddique, Trilochan Mohapatra, Jeffrey L. Bennetzen, Xun Xu, Xin Liu

**Affiliations:** 1 Center of Excellence in Genomics and Systems Biology, International Crops Research Institute for the Semi-Arid Tropics (ICRISAT), Hyderabad, India; 2 State Agricultural Biotechnology Centre, Centre for Crop and Food Innovation, Murdoch University, Murdoch, Western Australia Australia; 3 BGI-Qingdao, BGI-Shenzhen, Qingdao, China; 4 China National GeneBank, BGI-Shenzhen, Shenzhen, China; 5 College of Life Sciences, University of Chinese Academy of Sciences, Beijing, China; 6 Institute of Crop Germplasm Resources, Shandong Academy of Agricultural Sciences (SAAS), Jinan, China; 7 ICAR–Indian Institute of Pulses Research, Kanpur, India; 8 Genebank, ICRISAT, Hyderabad, India; 9 University of Georgia, Athens, GA USA; 10 BGI-Shenzhen, Shenzhen, China; 11 State Key Laboratory of Agricultural Genomics, BGI-Shenzhen, Shenzhen, China; 12 The UWA Institute of Agriculture, and School of Agriculture and Environment, The University of Western Australia, Perth, Western Australia Australia; 13 Biometrics and Statistics Unit, International Maize and Wheat Improvement Center (CIMMYT), Texcoco, Mexico; 14 Digital Agriculture Laboratory, Skolkovo Institute of Science and Technology, Moscow, Russia; 15 Queensland Alliance for Agriculture and Food Innovation, The University of Queensland, St Lucia, Queensland Australia; 16 International Rice Research Institute (IRRI), South-Asia Hub, ICRISAT, Hyderabad, India; 17 Laboratoire Ecologie Fonctionnelle et Environnement, Université de Toulouse, CNRS, Toulouse, France; 18 Institute for Genomic Diversity, Cornell University, Ithaca, NY USA; 19 Department of Plant Sciences, University of Saskatchewan, Saskatoon, Saskatchewan Canada; 20 ICAR–Indian Agricultural Research Institute (IARI), New Delhi, India; 21 Rajmata Vijayaraje Scindia Krishi Vishwa Vidyalaya, Gwalior, India; 22 Junagadh Agricultural University, Junagadh, India; 23 Rajasthan Agricultural Research Institute (RARI), Durgapura, India; 24 Department of Agronomy and Horticulture, University of Nebraska–Lincoln, Lincoln, NE USA; 25 DIADE (Diversity-Adaptation-Development of Plants), Université de Montpellier, Institut de Recherche pour le Développement (IRD), Montpellier, France; 26 International Centre for Agricultural Research in the Dry Areas (ICARDA), Cairo, Egypt; 27 International Centre for Agricultural Research in the Dry Areas (ICARDA), Rabat, Morocco; 28 Rani Lakshmi Bai Central Agricultural University, Jhansi, India; 29 National Institute of Plant Genome Research, New Delhi, India; 30 Department of Statistics, University of Nebraska–Lincoln, Lincoln, NE USA; 31 School of Plant Sciences, University of Arizona, Tucson, AZ USA; 32 Department of Plant and Soil Science, University of Vermont, Burlington, VT USA; 33 University of Calcutta, Kolkata, India; 34 Guangdong Provincial Academician Workstation of BGI Synthetic Genomics, BGI-Shenzhen, Shenzhen, China; 35 Division of Plant Sciences, University of Missouri, Columbia, MO USA; 36 James D. Watson Institute of Genome Science, Hangzhou, China; 37 Indian Council of Agricultural Research (ICAR), New Delhi, India; 38 Department of Genetics, University of Georgia, Athens, USA; 39 Guangdong Provincial Key Laboratory of Genome Read and Write, BGI-Shenzhen, Shenzhen, China; 40 BGI-Beijing, BGI-Shenzhen, Beijing, China; 41 BGI-Fuyang, BGI-Shenzhen, Fuyang, China

**Keywords:** Structural variation, Agricultural genetics, Plant breeding, Natural variation in plants, Plant breeding

## Abstract

Zero hunger and good health could be realized by 2030 through effective conservation, characterization and utilization of germplasm resources^1^. So far, few chickpea (*Cicer* *arietinum*) germplasm accessions have been characterized at the genome sequence level^2^. Here we present a detailed map of variation in 3,171 cultivated and 195 wild accessions to provide publicly available resources for chickpea genomics research and breeding. We constructed a chickpea pan-genome to describe genomic diversity across cultivated chickpea and its wild progenitor accessions. A divergence tree using genes present in around 80% of individuals in one species allowed us to estimate the divergence of *Cicer* over the last 21 million years. Our analysis found chromosomal segments and genes that show signatures of selection during domestication, migration and improvement. The chromosomal locations of deleterious mutations responsible for limited genetic diversity and decreased fitness were identified in elite germplasm. We identified superior haplotypes for improvement-related traits in landraces that can be introgressed into elite breeding lines through haplotype-based breeding, and found targets for purging deleterious alleles through genomics-assisted breeding and/or gene editing. Finally, we propose three crop breeding strategies based on genomic prediction to enhance crop productivity for 16 traits while avoiding the erosion of genetic diversity through optimal contribution selection (OCS)-based pre-breeding. The predicted performance for 100-seed weight, an important yield-related trait, increased by up to 23% and 12% with OCS- and haplotype-based genomic approaches, respectively.

## Abstract (web summary)

Whole-genome sequencing of 3,171 cultivated and 195 wild chickpea accessions is used to construct a chickpea pan-genome, providing insight into chickpea evolution and enabling breeding strategies that could improve crop productivity.

## Main

Pulses are an important crop commodity providing protein for human health. Worldwide pulse productivity has been stagnant for the last five decades, contributing to low per-capita availability of these foods and high levels of malnutrition in developing countries^3^. Chickpea (*Cicer arietinum* L.) production ranks third among pulses, and chickpea is cultivated in more than 50 countries, especially in South Asia and sub-Saharan Africa. As it is an important source of protein, dietary fibre and micronutrients, chickpea is key to nutritional security. More than 80,000 chickpea germplasm accessions are being conserved in 30 genebanks across the world^4^, but only a few have been used for chickpea improvement^2^.

Germplasm sequencing efforts in some crop plants have provided insights into the global distribution of genetic variation^5^; how this diversity has been shaped by the genetic bottlenecks associated with domestication^6^ and by the effects of selective breeding^7^; and, finally, how we can link this genetic variation to phenotypic diversity^2^ for breeding applications. Haplotype maps developed using whole-genome sequencing (WGS) data have helped to determine the percentage of the constrained genome and detect deleterious mutations that can be purged for accelerated breeding^8,9^. Furthermore, sequencing and genotyping of a germplasm collection allows better conservation and management in genebanks^5,10^.

On the basis of WGS of 3,366 chickpea germplasm accessions, we report here a rich map of the genetic variation in chickpea. We provide a chickpea pan-genome and offer insights into species divergence, the migration of the cultigen (*C. arietinum*), rare allele burden and fitness loss in chickpea. We propose three genomic breeding approaches—haplotype-based breeding, genomic prediction and OCS—for developing tailor-made high-yielding and climate-resilient chickpea varieties.

**Extended Data Fig. 1:**
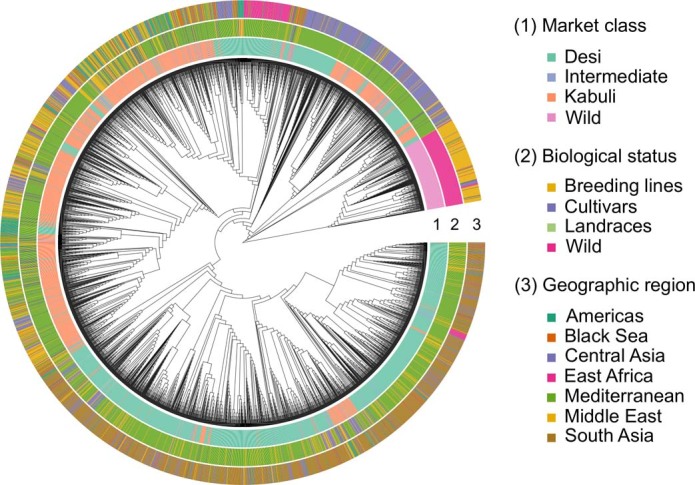
Phylogeny-based clustering. Phylogenetic tree represents clustering of individuals, represented through respective tracks (from inside to outside), Track 1: Market class; Track 2: Biological status; and Track 3: Geographical regions. A clear outgroup of wild accessions is observed.

**Extended Data Fig. 2:**
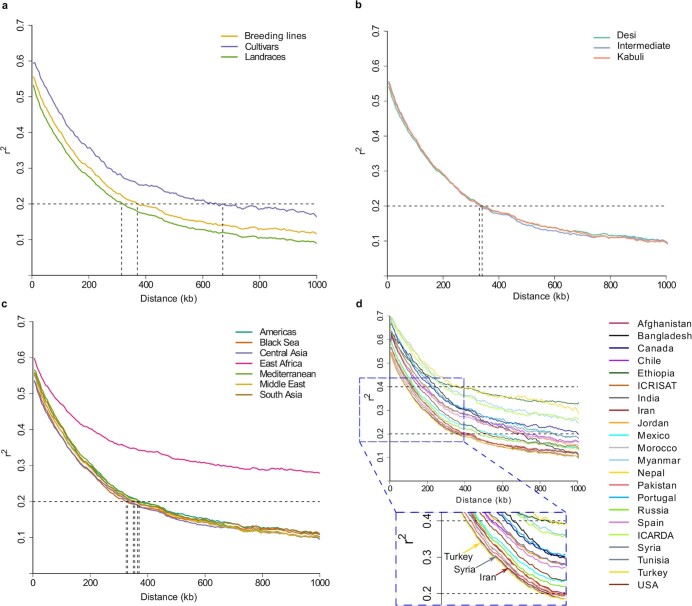
Linkage disequilibrium decay observed among cultivated chickpea genotypes. (a) Rapid LD decay was observed in landraces (315 kb) based biological status followed by breeding lines (370 kb) and cultivars (670 kb). (b) Similar LD decay rate was observed among population based on market class, namely desi (340 kb), intermediate (330 kb) and kabuli (330 kb). (c) Among seven geographic populations, genotypes from Black Sea (352 kb) had lowest rate of LD decay followed by Central Asia (330 kb), Middle East (350 kb), South Asia (355 kb), Mediterranean (365 kb) and Americas (370 kb). The population East Africa had much slower LD decay compared to other population based geographic regions. (d) Cultivated accessions from countries Turkey (306.51 kb), Syria (316.22 kb) and Iran (320.61 kb) had more rapid decline of LD decay compared to cultivated accessions from other countries, indicating more recombination events and haplotype diversity/number.

We sequenced 3,366 chickpea germplasm lines, including 3,171 cultivated and 195 wild accessions at an average coverage of around 12× (Methods, Extended Data Fig. 1, Supplementary Data 1 Tables 1, 2). Alignment of WGS data to the CDC Frontier reference genome^11^ identified 3.94 million and 19.57 million single-nucleotide polymorphisms (SNPs) in 3,171 cultivated and 195 wild accessions, respectively (Extended Data Table 1, Supplementary Data 1 Tables 3–7, Supplementary Notes). This SNP dataset was used to assess linkage disequilibrium (LD) decay (Supplementary Data 2 Tables 1, 2, Extended Data Fig. 2, Supplementary Notes) and identify private and population-enriched SNPs (Supplementary Data 3 Tables 1–4, Supplementary Notes). These private and population-enriched SNPs suggest rapid adaptation and can enhance the genetic foundation in the elite gene pool.

## Pan-genome

We developed a chickpea pan-genome (592.58 Mb) using an iterative mapping and assembly approach by combining the CDC Frontier reference genome, an additional 2.93 Mb from a desi genome (ICC 4958)^12^, 3.70 Mb from a *Cicer reticulatum* genome^13^ and 53.66 Mb from de-novo-assembled sequences from cultivated (48.38 Mb; 3,171) and *C. reticulatum* (5.28 Mb; 28) accessions (Supplementary Data 4 Table 1). Although similar pan-genome studies have been conducted in other crops, including rice^5,14^, soybean^15^ and *Brassica oleracea*^16^, our pan-genome comprises more than 3,000 individuals.

**Fig. 1:**
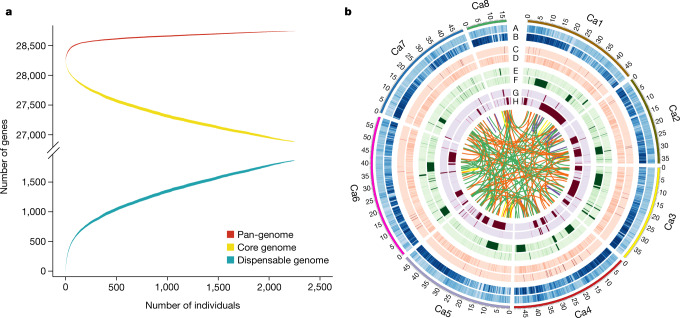
Global chickpea genetic variations. **a**, The chickpea pan-genome. Modelling analysis of the pan-genome and core genome shows an increase and decrease in the number of genes with each added genotype, indicating that the pan-genome is a closed pan-genome. The thickness of the curves represents the 99% confidence interval. **b**, Circos diagram illustrating the variation density among chickpea lines. Overall, higher numbers of variations were observed among wild accessions. Tracks indicate SNP density among cultivated (A) and wild (B), insertion density among cultivated (C) and *C. reticulatum* (D), deletion density among cultivated (E) and *C. reticulatum* (F), and inversion density among cultivated (G) and *C. reticulatum* (H). Links represent inter- and intra-chromosomal translocations. Yellow (cultivated) and purple (*C. reticulatum*) denote intra-chromosomal translocations, whereas orange (cultivated) and green (*C. reticulatum*) represent inter-chromosomal translocations. Source data

A total of 29,870 genes (1,601 additional gene models) were identified, of which 1,582 were to our knowledge novel compared to previously reported genes^11^. Gene ontology (GO) annotations identified genes that encode response to oxidative stress, response to stimulus, heat shock protein, cellular response to acidic pH and response to cold (Supplementary Data 4 Tables 2, 3), suggesting a possible role in adaptation. The modelling analysis curve eventually reaching saturation suggested that the pan-genome is closed, in concurrence with other plant pan-genomes^14,16^ (Fig. 1a). N50, a widely used metric to assess the quality of an assembly, is the length of the shortest contig for which larger and equal size contigs cover 50% of the total assembly. The N50 values for sequences from de-novo-assembled cultivated and *C. reticulatum* accessions, *C. reticulatum* and the desi genome were 2.61 kb, 1.30 kb, 1.78 kb and 1.76 kb, respectively, whereas the average gene length was 4.72 kb, 1.09 kb, 1.09 kb and 0.98 kb (Supplementary Data 4 Table 1). This pan-genome was further used to assess the effect of presence–absence variations on protein-coding genes (Supplementary Data 4 Table 4, Supplementary Notes).

Cultivated (2,258) and *C. reticulatum* (22) accessions with a coverage of greater than 10× were analysed to discover structural variations, including insertions (139,483), deletions (47,882), inversions (61,171), intra-chromosomal translocations (417) and inter-chromosomal translocations (2,410) in cultivated and 287,854 insertions, 67,351 deletions, 58,070 inversions, 446 intra-chromosomal translocations and 2,066 inter-chromosomal translocations among *C. reticulatum* accessions as compared to the CDC Frontier genome^11^ (Fig. 1b, Extended Data Table 1, Supplementary Data 5 Table 1, Supplementary Notes). More structural variations in the *C. reticulatum* accessions were expected because of their high divergence from cultivated chickpea. We further identified 793 gene-gain copy number variants (CNVs) and 209 gene-loss CNVs incultivated accessions, and 643 gene-gain and 247 gene-loss CNVs in *C. reticulatum* accessions (Supplementary Data 5 Tables 2, 3).

## Species divergence and migration

**Extended Data Fig. 3:**
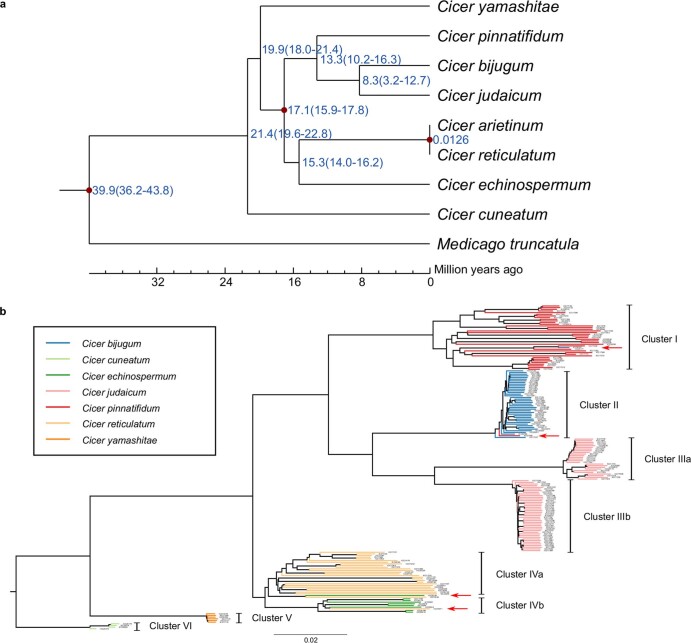
*Cicer* species evolution. (a) Speciation and divergence time for eight species in the genus *Cicer*. The maximum likelihood phylogenetic tree showed clear out-grouping of *C. cuneatum* from the other *Cicer* species and *C. reticulatum* being nearest to the cultivated chickpea species (*C. arietinum*). Three time-calibration points (0.007- 0.013 Ma for *C. reticulatum*-*C. arietinum*, 12.2-17.4 Ma for *C. arietinum*-*C. pinnatifidum*, and 30.0-54.0 Ma for *C. arietinum- M. truncatula*) were used for estimating divergence time. The nearest wild species (*C. reticulatum* and *C. echinospermum*) related to the cultivated *C. arietinum* were estimated to be diverged from other *Cicer* species around ~15.3 (14.0-16.2) Ma. (b) Genetic diversity among wild species accessions. Phylogenetic tree constructed based on SNPs grouped 195 wild species accessions into six clusters. A clear grouping for accessions of *C. judiacum*, *C. yamashitae* and *C. cuneatum* was observed in Cluster III, Cluster V and Cluster VI, respectively. However, ICC 20168 (one *C. pinnatifidum* accession; red colour) grouped along with *C. bijugum* accessions in Cluster II; similarly, ICC20167 (one *C. bijugum* accession; blue colour) grouped along with *C. pinnatifidum* accessions in Cluster I. Cluster III and Cluster IV were divided into two sub-clusters each, in which both sub-clusters of Cluster III possessed all accessions of *C. judiacum*. In Cluster IVa we observed grouping of all *C. reticulatum* accessions except one *C. echinospermum* accession (ICC 20192; green colour); similarly, in Cluster IVb one accession of *C. reticulatum* (ICC 73071; golden-yellow colour) grouped along with *C. echinospermum* accessions.

To understand speciation and estimate species divergence time in the eight *Cicer* species analysed here, single-copy genes identified using ‘fabales’ genes from the BUSCO^17^ database were used to carry out homologue-based gene annotation in preliminary genome assemblies, the CDC Frontier^11^ and *Medicago truncatula*^18^. Using these single-copy genes, *Cicer cuneatum* was estimated to have diverged from other *Cicer* species around 21.4 (19.6–22.8) million years ago (Ma) (Extended Data Fig. 3a, Supplementary Notes), about the time that Arabia collided with Asia, and a time when ‘Rand Flora’ taxa like *Cicer* may have migrated from Africa into Southwest Asian habitats^19^. *C. reticulatum* and *Cicer echinospermum* were estimated to have diverged around 15.3 (14.0 to 16.2) Ma, which is higher than previous estimates and might be influenced by: (i) wild accessions conserved at the International Crops Research Institute for the Semi-Arid Tropics (ICRISAT) representing only some populations of these species, when recent work has shown that only some *C. echinospermum* populations are cross-compatible with *C. arietinum*; and (ii) introgression from *C. echinospermum* into cultivated chickpea, which is widespread in Australian and North American breeding lines, and is also likely to have occurred in International Center for Agricultural Research in the Dry Areas (ICARDA) lines.

Phylogenetic analysis grouped all 195 wild accessions into 6 clusters (Clusters I–VI) (Extended Data Fig. 3b, Supplementary Notes). Cluster IVa included all *C. reticulatum* and one *C. echinospermum* (ICC 20192; green colour), whereas cluster IVb included all *C. echinospermum* and one *C. reticulatum* (ICC 73071; golden-yellow colour). Similarly, one *Cicer pinnatifidum* (ICC 20168; red colour) was grouped with the *Cicer bijugum* accessions in cluster II, and one *C. bijugum* (ICC 20167; blue colour) was grouped with *C. pinnatifidum* accessions in cluster I. These are two cross-compatible species. Spontaneous hybridization might have occurred in nature. In terms of post-species divergence, a homologue (*Ca_25684*) of *SHATTERPROOF2* (also known as Agamous-like MADS-box protein (*AGL5*)), which is responsible for seed dispersal, was analysed for haplotypic variation (Supplementary Notes). We found an association of the ‘C’ allele with low or minimal shattering in cultivated species, as seen at the low shattering allele (‘C’) on chromosome 5 at position 1,022,962 of the orthologue in common bean^20^.

**Fig. 2:**
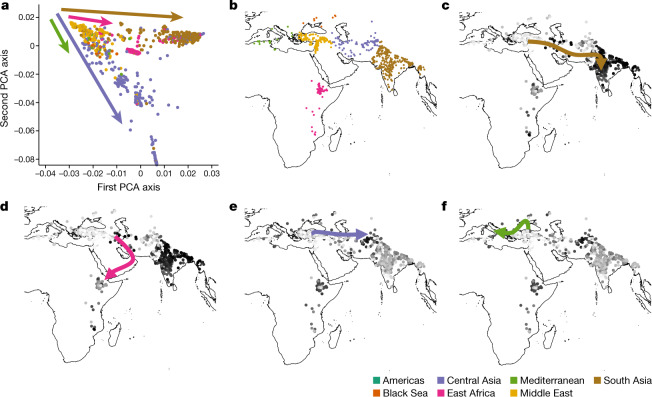
Insights into chickpea migration. **a**–**f**, The PCA based on geographic origin suggests two paths of diffusion (**a**, **b**). The first path illustrates a diffusion to South Asia (**c**) and East Africa in parallel (**d**). The second path suggests a diffusion to Central Asia (**e**) together with the Mediterranean region (**f**).

**Extended Data Fig. 4:**
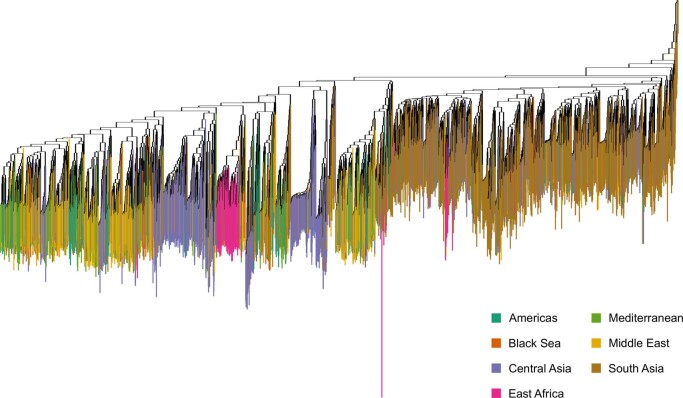
Phylogenetic tree based on *F*_ST_. Accessions from Mediterranean region, Middle East, Americas and Black Sea regions were clustered together, and South Asia as a separate cluster.

**Extended Data Fig. 5:**
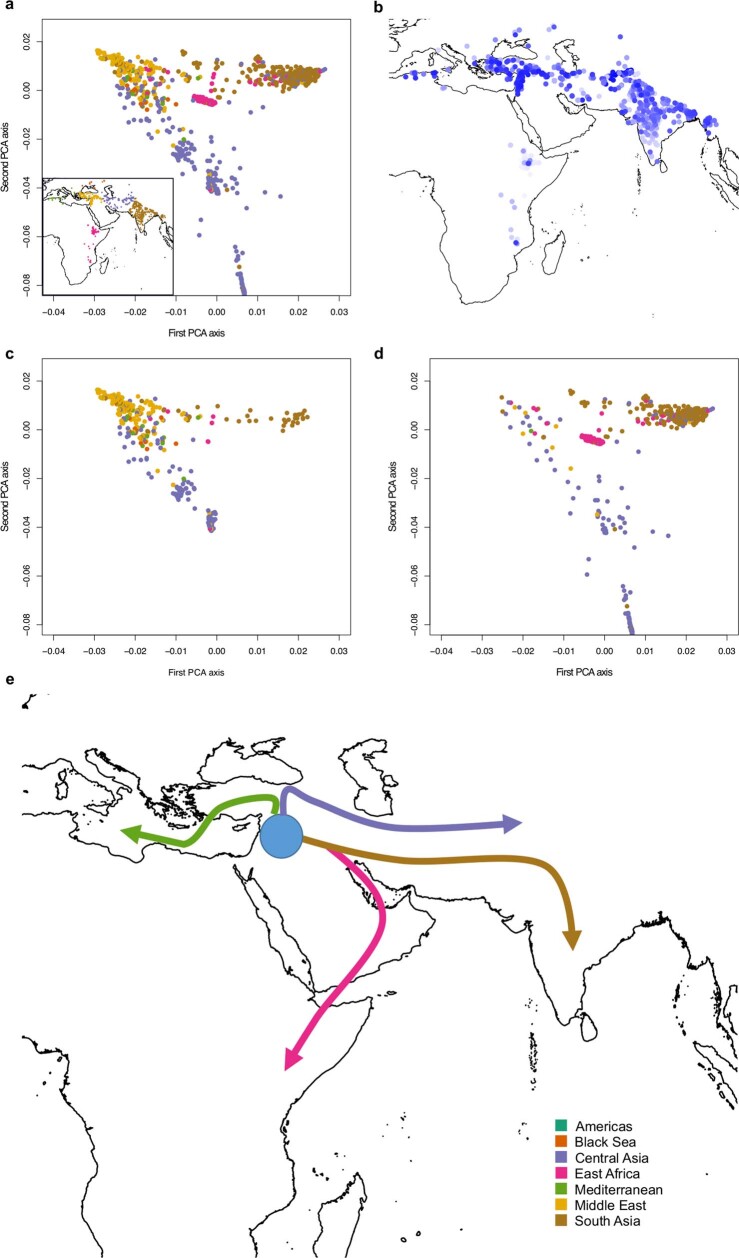
Relationship route of chickpea diffusion and seed morphology. (a) PCA analysis for landraces. (b) Distance to the most extreme cultivated sample (closest to wild relatives) were plotted on the map. (c, d) For landraces with large seed morphology (kabuli; c) and small seed morphology (desi; d) indicated that small seed was mainly found in East-Asia, South-East Asia and Africa. These suggest large and small seeds were selected independently during chickpea diffusion of agriculture. (e) PCA results summarised a Central Asian diffusion alongside a Mediterranean diffusion, and a South Asian diffusion associated with the diffusion to East Africa.

The neighbour-joining tree grouped most South Asian accessions with no distinct clustering for other geographic origins (Extended Data Fig. 4). Our principal component analysis (PCA) of accessions suggests two paths of diffusion or migration of chickpea from the centre of origin in the Fertile Crescent: one path indicates diffusion to South Asia and East Africa, and the other suggests diffusion to the Mediterranean region (probably through Turkey) as well as to the Black Sea and Central Asia (up to Afghanistan) (Fig. 2a–f, Extended Data Fig. 5). This diffusion translated into a pattern of nucleotide diversity (*π*), among accessions from Central Asia (4.74 × 10^−4^) and South Asia (3.62 × 10^−4^) (Supplementary Data 6 Table 1), which is consistent with earlier reports^2^. Pairwise fixation index (*F*_ST_) estimations further supported these findings (Supplementary Data 6 Table 2, Supplementary Notes).

## Domestication and breeding bottlenecks

**Extended Data Fig. 6:**
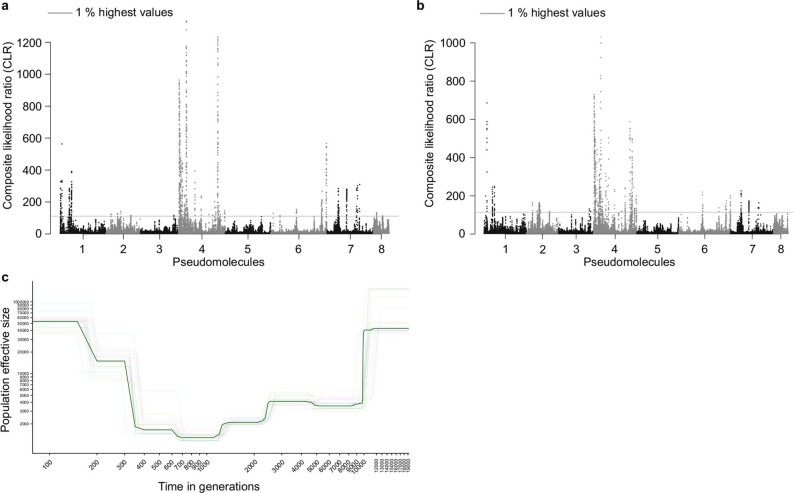
Composite likelihood ratio values along the chickpea genome and inference of past evolution of effective size. The composite likelihood ratio for chromosomes 1 to 8 on the x axis is computed for two random subsets of 251 individuals: subset 1 (a) and subset 2 (b). Horizontal grey line shows the threshold above which the highest 1% CLR values are found. (c) Using sequentially markovian coalescent as implemented in SMC++ (Terhorst et al. 2017), we reconstructed the past history of effective size for 20 sets of 150 randomly chosen cultivated genotypes (thin lines). We computed at each time point the median of the estimated histories and plotted it (bold lines). Focus was made for the plotting on timeframe 100 – 20,000 generations ago. Both x and y axes are log-scaled.

Our analysis indicates that chickpea experienced a strong bottleneck beginning around 10,000 years ago. The population size reaching its minimum around 1,000 years ago, followed by a very strong expansion of the population within the last 400 years (Extended Data Fig. 6), suggest a strong recent expansion of chickpea agriculture. One consequence of this bottleneck is shown by the higher *π* in *C. reticulatum* (2.20 × 10^–3^) relative to cultivated accessions (Extended Data Table 1, Supplementary Data 6 Table 1).

**Extended Data Fig. 7:**
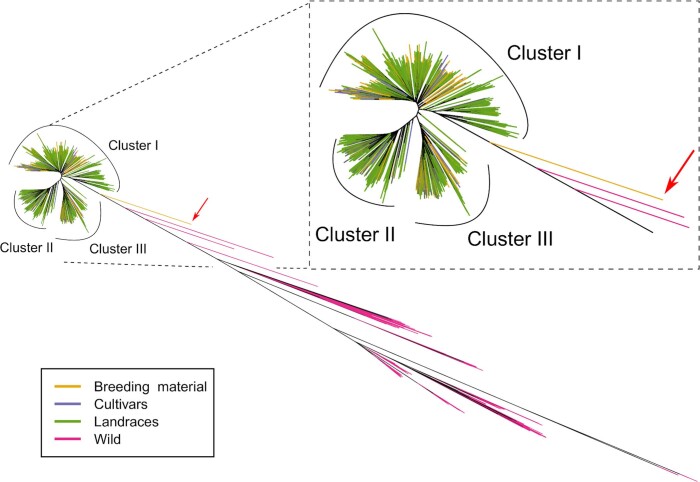
Neighbour-joining trees constructed using SNPs present on the pseudomolecules indicates a clear out-grouping of wild species accessions from cultivated accessions. The cultivated accessions formed three distinct clusters. One landrace from East Africa (ICC 16369) (red arrow) grouped together with wild species accessions.

Genetic relationship analysis between cultivated and wild chickpea showed that one cultivated accession (ICC 16369) from East Africa was grouped with wild chickpea (Extended Data Fig. 7). This same genotype also showed the presence of the ‘T’ allele, specific to wild species in *SHATTERPROOF2*, suggesting that ICC 16369 has been mislabelled as belonging to the cultivated chickpea (Supplementary Data 7).

To detect selection sweeps, we pinpointed 18 fragments in cultivated chickpea using the composite likelihood ratio (CLR) (Extended Data Fig. 6, Supplementary Data 6 Tables 8, 9). Combined analysis with reduction of diversity (ROD), *F*_ST_ and Tajima’s *D* identified genomic regions for *C. reticulatum* (immediate wild species progenitor) versus landraces (2,899; 42,148 kb), landraces versus breeding lines (191; 4,360 kb) and breeding lines versus cultivars (14; 404 kb) that might have undergone selection during domestication and breeding (Supplementary Data 6 Tables 3–6, Supplementary Notes, 10.6084/m9.figshare.15015327). We identified 35 regions (222 kb) common between *C. reticulatum* versus landraces and landraces versus breeding lines, and similarly one region (4 kb) between landraces versus breeding lines and breeding lines versus cultivars. Furthermore, we identified a total of 37 unique potential genes in these 36 regions that may have a role in the adaptation of chickpea during migration to different environments by regulating flowering time and plant growth (Supplementary Data 6 Table 7). For example, *FLP2* (flower development and vegetative to reproductive phase transition of meristem), *LRP1* (root growth), *PIP5KL1* (signalling pathways for survival and T cell metabolism) and *MYB12* (flavonoid biosynthesis) are some key genes we pinpointed that are critical for plant growth, metabolic pathways and adaptation in changing environments.

**Extended Data Fig. 8:**
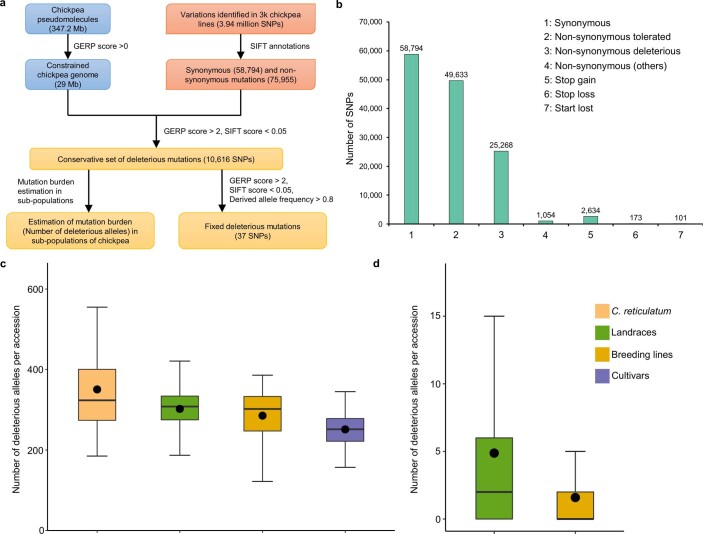
Genetic loads in chickpea. a) A snapshot of steps and parameters used to estimate the mutation burden and fixed deleterious alleles. b) Variant annotation using SIFT revealed higher non-synonymous mutations, of which non-synonymous deleterious variants were used to identify deleterious mutations. c) Mutation burden analysis indicated a 17.88% decrease (two-tailed Welch’s t-test; *t* = 2.525, *df* = 27, *p* = 0.01772, CI = 95%) in mutation burden in cultivated (*n* = 2987) as compared to progenitor (*C. reticulatum; n* = 28). d) Mutation burden for genomic regions under selection showed that landraces (*n* = 2439) contained 206.91% higher (two-tailed Welch’s t-test, *t* = −17.087, *df* = 1645, *p* = 2.195676 × 10^−60^, CI = 95%) deleterious mutations than breeding lines (*n* = 396). The black solid dots in box plots represent mean values for the respective population. Each of the box plots shows the upper and lower whisker, the 25% and 75% quartiles, the median (as solid line) and the mean (black dot) Source data

We used genomic evolutionary rate profiling (GERP) analysis to identify 29 Mb (8.36%) genomic regions as evolutionarily constrained (GERP score of greater than 0), indicating purifying selection (Extended Data Fig. 8a). Using constrained genome, sorting intolerant from tolerant^21^ (SIFT) score (less than 0.05) and GERP (greater than 2), 10,616 non-synonymous SNPs were identified as candidate deleterious mutations (Extended Data Fig. 8b). Using the derived allele frequency (DAF) spectrum, we selected 37 non-synonymous deleterious mutations (SIFT < 0.05; GERP > 2; DAF > 0.8) in 36 genes (Supplementary Data 8 Tables 1–4), as fixed that have not been purged through traditional breeding. Detailed analysis indicated a higher (17.88%, *P* = 0.01772) abundance of deleterious alleles in the wild progenitor (*C. reticulatum*) than in cultivated accessions (Extended Data Fig. 8c). Furthermore, the mutation burden for genomic regions under selection suggested that the number of deleterious mutations in landraces was approximately twofold that in breeding lines (206.91%; *P* = 2.195676 × 10^−60^) (Extended Data Fig. 8d). To increase the fitness of cultivated chickpea, these deleterious alleles are potential targets for genomics-assisted breeding and genome editing.

## Superior haplotypes for key traits

**Extended Data Fig. 9:**
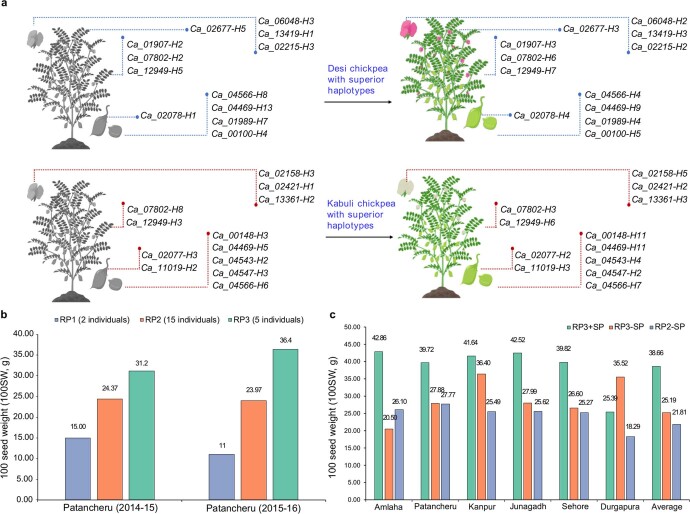
Towards developing tailored chickpea with superior haplotypes for yield and related traits. (a) Representative desi and kabuli chickpea plant (on left) carrying inferior haplotype combination for key traits including 100 seed weight (100SW), days to maturity (DM), plant height (PLHT), pods per plant (PPP), and plot yield (PY). Target desi and kabuli chickpea plant (on right) carrying superior haplotype for 100SW, DM, PLHT, PPP and PY. New breeding lines can be developed by introgressing the superior haplotype combination through haplotype-based breeding. (b) Comparison of average performance among RP1 vs RP2 vs RP3 varieties for 100SW at Patancheru location. An increase in 100SW between the varieties of RP1 vs RP3 was observed, whereas no differences were observed in the case of RP1 vs RP2 and RP2 vs RP3 varieties (datasets of ICRISAT 2014-15 and 2015-16). RP1 indicates chickpea varieties released before 1993, RP2 indicates chickpea varieties released between 1993-2002 and RP3 indicates chickpea varieties released after 2002. (c) Comparison of RP2 and RP3 varieties for 100SW (with and without superior haplotypes) for six locations. A difference between lines carrying the superior haplotypes (RP3+SP) for 100SW was observed in comparison to those which did not (RP3-SP and RP2-SP) except for the Durgapura location. However, marked differences were also observed between the RP3-SP and RP2-SP lines, except for Patancheru and Amlaha locations. RP3+SP indicates RP3 varieties with superior haplotypes, RP3-SP indicates RP3 varieties without 100SW superior haplotype and RP-SP indicates RP2 varieties without 100SW superior haplotype. RP2 indicates chickpea varieties released between 1993-2002 and RP3 indicates chickpea varieties released after 2002.

We used 3.94 million SNPs and phenotyping data for 16 traits on 2,980 cultivated genotypes to identify 205 SNPs associated with 11 traits (Methods, Supplementary Data 9 Table 1, Supplementary Notes). Of the 205 associated SNPs, 152 were present in 79 unique genes with potential roles in controlling seed size and development. Analysis of these genes across cultivated genotypes identified 350 haplotypes (Supplementary Data 9 Tables 2–4, Supplementary Notes). Using 19.10 million haplo–pheno combinations, we identified 24 consistent and stable superior haplotypes for 20 genes (Supplementary Data 9 Tables 5–7, Extended Data Fig. 9a). This analysis revealed that the majority of breeding lines (80%) lacked superior haplotypes that are present in the landraces. We validated superior haplotypes by using historical data on 129 chickpea varieties released between 1948 and 2012 (Extended Data Fig. 9b, c). Finally, we identified 56 lines as potential donors for introducing superior haplotypes in breeding (Supplementary Data 9 Tables 8–10).

## Enriching the genetic base

We combined OCS^22^ with a mate allocation method that takes into account genetic gain and genetic diversity as a guide for potential future chickpea pre-breeding programmes or ‘evolving gene banks’^22,23^ (Supplementary Notes). With a price bonus for earliness and for large seeds, we chose 274 (9.4%) unique genotypes for 325 matings from the 2,898 available genotypes, divided among desi (190), kabuli (120) and intermediate (15), using MateSel^24^ (Supplementary Data 10 Table 1).

**Fig. 3:**
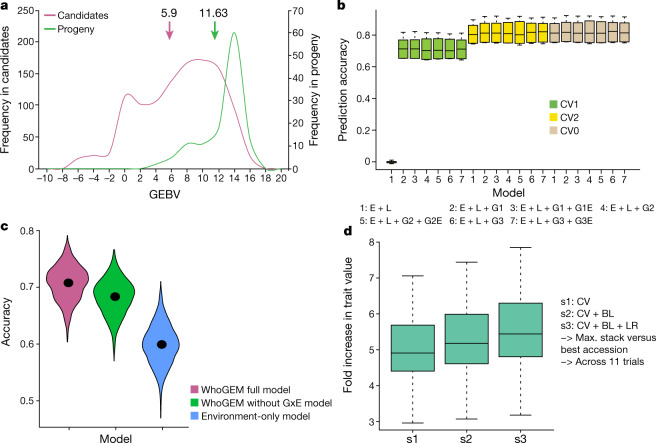
An example of the use of four genomic breeding strategies for improving 100SW. **a**, Mean GEBV and total genetic values predict a 23% increase in one generation for 100SW in kabuli candidates. **b**, Genomic-enabled predictions using Bayesian generalized linear regression (BGLR) on three cross-validation schemes provided the highest mean prediction accuracy with scheme CV0 (*n* = 2,980 cultivated accessions). **c**, A general linear model using the WhoGEM prediction machine provided the highest prediction accuracies for the WhoGEM full model (*n* = 1,500; 300 replicates of a fivefold cross-validation). In each violin plot, the black dot represents the mean. GxE, genotype and environment interaction. **d**, Haplotype-based local GEBVs that are suggested to provide a fivefold improvement in performance over the best accessions with the highest GEBV. The genotypes were classified into three different groups (cultivars (CV, *n* = 152), breeding lines (BL, *n* = 396) and landraces (LR, *n* = 2,439)). Each of the box plots shows the upper and lower whisker (indicated by dashed lines), the 25% and 75% quartiles and the median (as a solid line). Source data

**Extended Data Fig. 10:**
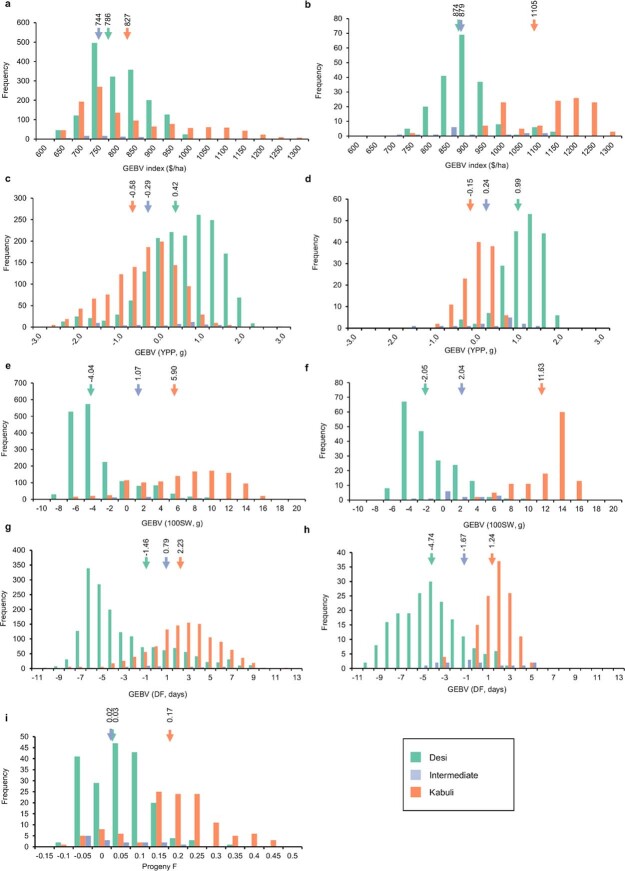
Response to OCS based on mating allocation for candidate parents and predicted cycle 1 progeny family means in economic index. Index increased from parents to cycle 1 progeny in the kabuli group by US$274/ha, and in the desi group by US$94/ha, and reflects the high value of large seeds in the kabuli group. Arrows indicate the population mean GEBVs for desi (green), kabuli (orange) and intermediate (blue) groups. (a) Response to selection for candidate parents. (b) Response to selection for predicted cycle 1 progeny family. (c, d) Response to OCS among desi, kabuli and Intermediate accessions is shown for genomic estimated breeding values (GEBVs) for yield per plant (YPP, g). YPP increased by 0.6 g (4.5%) above the average candidate parent YPP (13.2 g) in the desi group, and by 0.4 g (3.3%) above the average candidate parent yield (12.2 g) in the kabuli group. Arrows indicate the population mean GEBVs for desi (green), kabuli (orange) and intermediate (blue) groups. (c) candidate parents. (d) predicted cycle 1 progeny family. (e, f) Response to OCS for GEBVs for 100 seed weight (100SW, g). 100SW increased by 2.0 g (12.7%) above the average candidate parent 100SW (15.0 g) in the desi group, and by 5.7 g (22.9%) above the average candidate parent 100SW (24.9 g) in the kabuli group. Arrows indicate the population mean GEBVs for desi (green), kabuli (orange) and intermediate (blue) groups. (e) candidate parents. (f) predicted cycle 1 progeny family. (g, h) Response to OCS for GEBVs for days to flower (DF). DF decreased by 3.3 d (−4.7%) below the average candidate parent DF (68.6 d) in the desi group, and by 1.0 d (−1.4%) below the average candidate parent DF (72.3 d) in the kabuli group. Arrows indicate the population mean GEBVs for desi (green), kabuli (orange) and intermediate (blue) groups. (g) candidate parents. (h) predicted cycle 1 progeny family. (i) Predicted average inbreeding (F) in cycle 1 progeny in among desi, kabuli and intermediate accessions. Progeny inbreeding increased by 0.170 in the kabuli group, by 0.025 in the desi group, and by 0.015 in intermediate group. Arrows indicate the population mean GEBVs for desi (green), kabuli (orange) and intermediate (blue) groups.

The frequency distribution of predicted progeny index (mean of nine environments) values was bimodal. Higher predicted progeny index values were observed in kabuli as compared with desi. However, marked improvements were predicted in desi and kabuli, from candidate parents to predicted progenies (Extended Data Fig. 10a, b). The frequency distribution of predicted progeny genomic estimated breeding value (GEBV) for yield per plant (YPP) in desi (13.79 g) exceeded kabuli (12.65 g) and a higher response to selection was observed for desi (0.6 g; 4.3%) than for kabuli (0.4 g; 3.5%) (Extended Data Fig. 10c, d). For 100-seed weight (100SW), the mean 100SW of predicted progeny in kabuli (30.6 g) was almost twice that of desi (16.9 g), and the response to selection was three times higher for kabuli (5.7 g; 23%) than for desi (2.0 g; 13%) (Fig. 3a, Extended Data Fig. 10e, f). Kabuli progeny, with a later flowering time, did not respond to selection for earliness (−1.0 day) as rapidly as desi progeny (−3.3 days) (Extended Data Fig. 10g, h). These predicted responses to selection in the next cycle occurred with a relatively small increase in predicted progeny inbreeding in the desi (0.03) and intermediate (0.02), but a large increase in the kabuli (0.17) (Extended Data Fig. 10i, Supplementary Data 10 Table 2, Supplementary Notes).

## Breeding population improvement

We used different subsets of SNPs and phenotyping data on 16 traits across 12 combinations of year and location, following 3 genomic prediction approaches: (i) interaction of marker and environment covariates (*G* × *E*)^25^; (ii) implementation of the WhoGEM approach^26^; and (iii) a haplotype-based approach for estimating local GEBVs^27^.

In the first approach, 3 genomic relationship matrices with 223,119 (G1), 531,457 (G2) and 754,576 (G3) SNPs, and phenotyping data for 9 traits on 2,980 genotypes, were used to understand the variability explained within the groups and environments (Supplementary Data 10 Table 3). Overall, the environment (E) + genotype (L) + marker effects (G3) model for cross-validation scheme 0 (CV0; see ‘Prediction using the interaction of genomic and environmental covariates’ in Methods) produced the highest average correlation (0.719) for 100SW, and the E + L model returned the lowest value (0.031) for basal secondary branch (Supplementary Data 10 Table 4). For 100SW, genomic prediction accuracy varied from 0.611 (E + L + G3 + G3E) to 0.719 (E + L + G3) for CV1 and CV0, respectively (Fig. 3b).

**Extended Data Fig. 11:**
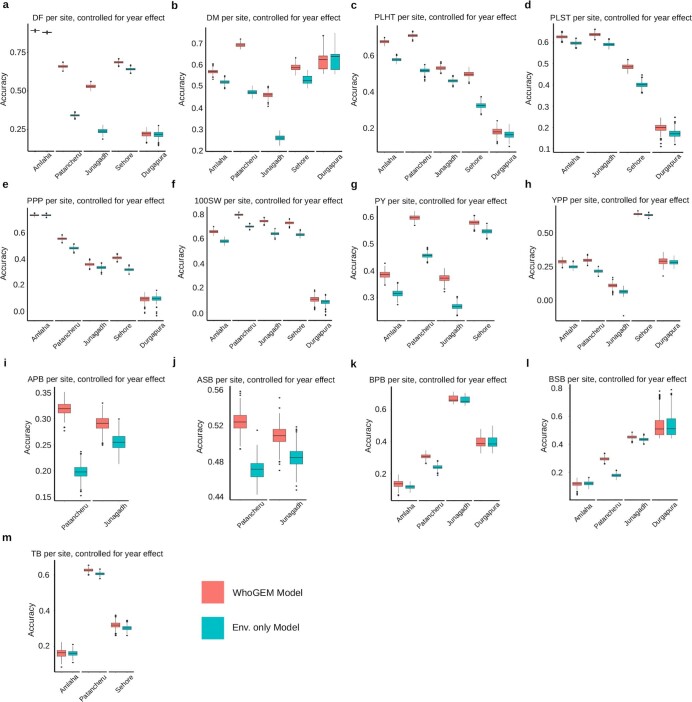
WhoGEM prediction accuracies for different traits in different sites. A general linear model was used for predicting performance in selected (with a geolocation) 1,318 cultivated chickpea accessions. At each site, 200 replicates of a fivefold cross-validation scheme are applied to estimate the accuracies of WhoGEM model (phenotype as a function of admixture components and market class) compared to environment-only model i.e. a model without genetic effects. Tests of WhoGEM significance are given by likelihood ratio tests between the WhoGEM-based models and the environment-only-based model. Phenology traits: (a) days to flowering (DF), (b) days to maturity (DM), (c) plant height (PLHT) and (d) plant stand (PLST); Production traits: (e) pods per plant (PPP), (f) 100 seed weight (100SW), (g) plot yield (PY) and (h) yield per plant (YPP) and Plant architecture traits: (i) apical primary branch (APB), (j) apical secondary branch (ASB), (k) basal primary branch (BPB), (l) basal secondary branch (BSB), and (m) tertiary branch (TB). Each of the box plots shows the upper and lower whisker, the 25% and 75% quartiles and the median (as solid line) of the fold change (*n* = 1,318 cultivated accessions). Source data

In the second approach, we used WhoGEM with 276,956 LD-pruned SNPs and phenotyping data for 9 traits on 1,318 genotypes (with GPS data). Prediction accuracies of the full model ranged from 0.25 to 0.91 (Supplementary Data 10 Table 5). Although the highest prediction accuracy was obtained for plot yield (0.914), this method was still efficient in predicting 100SW, with an accuracy of 0.599 (environment-only model) to 0.707 (WhoGEM full model) (Fig. 3c). Evidence for interactions between admixture components and the environment was presented for phenology, plant production and plant architecture traits (Extended Data Fig. 11a–m). The use of admixture components integrates the effects of demography (that is, gene flow and genetic drift) and artificial or natural selection to explain phenotypic variation with reasonable accuracy. This shows considerable potential to detect the accumulation of favourable admixture components from the wider genepool.

**Extended Data Fig. 12:**
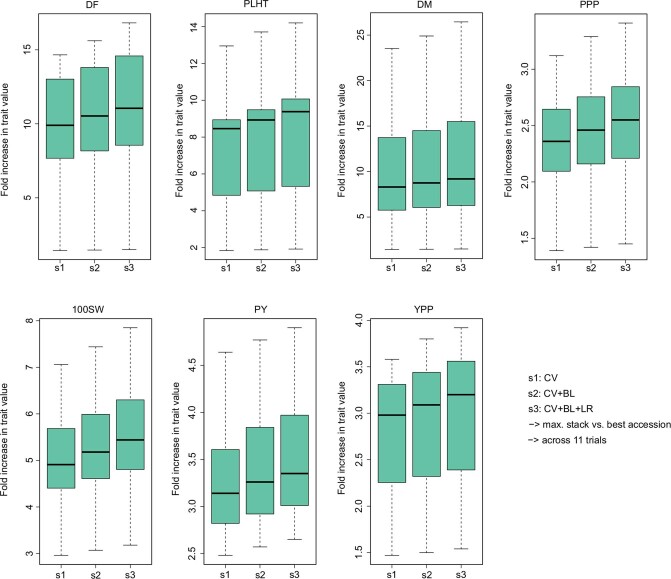
Assessment of trait improvement potential by stacking the superior haplotypes for target traits. The genotypes were classified into three different groups (cultivars (CV, *n* = 152); breeding lines (BL, *n* = 396) and landraces (LR, *n* = 2,439)) and these genotypes were grouped in three subgroups s1 (CV), s2 (CV+BL) and s3 (CV+BL+LR). Local GEBVs for haplotypes were calculated by firstly grouping SNP markers based on their pairwise linkage disequilibrium, and then summing up allele effects for each haplotype of each block. The best possible genotype for each trait was generated in silico by adding up the best haplotypes across the whole genome. This in silico genotype was then compared to the accession with the highest GEBV. Each of the box plots shows the upper and lower whisker (indicated by dashed lines), the 25% and 75% quartiles and the median (as solid line) Source data

In the third approach, 124,833 selected SNPs were used to construct LD blocks, called haplotypes. These SNPs and phenotyping data for 100SW and YPP for 2,980 genotypes were used to estimate local GEBVs for the haplotypes. The local GEBV analysis revealed substantial genetic potential in each subgroup for trait improvement (Extended Data Fig. 12). When comparing the best accessions with the highest GEBVs to the in silico genotypes that combined all haplotypes with the highest trait effect across the whole genome, the predicted performance increased by more than threefold for YPP and by more than fivefold for 100SW (Fig. 3d). Our results indicate that capturing novel alleles from landraces through a haplotype-based prediction approach could improve YPP or 100SW by 6–12% (Fig. 3d).

## Discussion

Our study reports global polymorphisms in chickpea by sequencing 3,366 germplasm accessions (3,171 cultivated and 195 wild). This analysis brings greater resolution to our understanding of the within-species diversity of *C. arietinum*. The chickpea pan-genome (592.58 Mb) developed from cultivated draft genomes^11,12^ and the *C. reticulatum* genome^13^, together with WGS data on cultivated and *C. reticulatum* accessions, provided insights into gene content variation across cultivated chickpea and its wild progenitor.

Although some studies based on chloroplast DNA^28^ and nuclear ribosomal DNA^29^ have been conducted to investigate the evolution and domestication of *Cicer* species in the past, their resolution was limited. Here, by using WGS data for a large number of individuals, we estimated the divergence time between chickpea and its closest progenitor species. Our study also provides opportunities to rectify misclassifications of accessions to the correct species and to determine whether chickpea seeds preserved in archaeological sites were wild or cultivated.

We identified selective sweeps and candidate genes under domestication and breeding that were responsible for reducing genetic diversity in the cultivated genepool. Most importantly, our study analysed genetic loads in *Cicer* species. Although selection and recombination have successfully purged many deleterious alleles, the current collection of breeding lines and cultivars still contains substantial genetic loads that affect crop fitness. Here, we have identified deleterious alleles for purging through genome-informed breeding and/or gene editing.

We identified numerous superior haplotypes for improvement-related traits in landraces, and used the concept of superior haplotypes by comparing the yield of the released varieties carrying superior versus regular haplotypes for yield-related traits^30^. Furthermore, we estimated prediction accuracies for agronomic traits using three genomic prediction approaches and provided a case study for 100SW, demonstrating that genomic prediction approaches have great potential for enhancing crop productivity. We suggest using haplotype mining and genomic prediction approaches in chickpea and other crops to provide climate resilience and improved nutrition to meet future worldwide demand.

## Methods

### Germplasm sequencing and variant calling

We performed WGS of 2,967 *Cicer* accessions from a global composite collection^4^ using the HiSeq2500 at the Center of Excellence in Genomics and Systems Biology, ICRISAT. By including sequence data of 399 lines from an earlier study^2^, we analysed 3,366 accessions (3,171 cultivated and 195 wild species accessions) altogether (Supplementary Notes).

We aligned sequencing data from the 3,366 chickpea accessions to the reference genome of CDC Frontier^11^, using BWA-MEM^31^ v.0.7.15. SNP calling was performed using GATK^32^ v.3.7 as per GATK best practices for SNP calling, thus creating the base SNP set. We defined two other SNP sets: (i) Set-A: only SNPs with <30% missing call, and biallelic calls, and (ii) Set-B: SNPs with less than 30% missing calls, biallelic calls, and LD-pruned using PLINK^33^ v.1.90 (“--indep-pairphase 50 10 0.2” parameter). Set-B SNPs were only used to depict the population genetic structure.

### Private and population-enriched SNPs

To determine the private and population-specific SNPs, the frequency of alleles within a given population was determined using VariantsToTable^34^ of GATK v3.8.1. We defined ‘private alleles’ as those present in at least four accessions within a population and absent in other populations, and ‘population-enriched alleles’ as those present in a given population (≥20%) and less frequent in other populations^5^ (≤2%).

### LD decay, diversity and *F*_ST_

LD decay was determined using the software PopLDdecay^35^ v.3.29 with the parameter “-MaxDist 1000”. Nucleotide diversity (*π*) was calculated from a 100-kb sliding window with a 10-kb step using VCFtools^36^ v.0.1.13. The average of all valid windows was considered the population genetic diversity. The fixation index (*F*_ST_) was calculated from 100-kb non-overlapping windows using VCFtools. The global weighted *F*_ST_ was used to measure the differentiation of populations.

### Construction of a pan-genome

The chickpea draft genome of CDC Frontier^11^ (a kabuli variety; considered as the foundation genome) together with ICC 4958^12,37^ (a desi genome sequence), a *C. reticulatum* genome^13^, and de-novo-assembled sequences from 3,171 cultivated and 28 *C. reticulatum* accessions were used to guide the assembly of the chickpea pan-genome using a conservative approach^38^. Following the alignment of reads from each accession to the reference, unmapped and dangling mapped read pairs were extracted using SAMTools^39^ v.1.2 based on the FLAG field. The extracted reads were de-novo-assembled using MEGAHIT^40^ v.1.2.9 with default parameters. To identify possible redundancies among assembled contigs that were already present in the foundation genome, the assembled contigs were aligned to the foundation genome using NUCmer^41^ v.4.0.0beta2 with the parameters “-l 20 -c 65” and the alignments with length ≥ 500 bp and identity of greater than 80% were extracted to be added into the intermediate pan-genome. The processes were performed one by one: ICC 4958, de-novo-assembled sequences from 3,171 cultivated accessions, the *C. reticulatum* genome, and de-novo-assembled sequences from 28 *C. reticulatum* accessions. Further, to identify redundancy among the ‘novel’ sequences, all-versus-all alignment was performed using CD-HIT^42^ v.4.81. The same process was performed for the next iteration until no sequence was left. Finally, we removed the potential containments from vectors, bacteria, viruses, animals, fungi and organelle sequences using BLASTN^43^ v.2.2.31 to the corresponding NT databases and obtained the final pan-genome. As a result, the CDC Frontier genome^11^ and novel assembled sequences were combined to construct the chickpea pan-genome.

### Structural and copy number variations

A total of 2,258 cultivated and 22 *C. reticulatum* accessions (with sequence depth of greater than 10×) were used to identify structural variations against the reference genome of CDC Frontier^11^, such as large insertions, deletions, inversions, and intra- and inter-chromosomal translocations. The insertions, deletions and inversions were identified using a dual calling strategy through BreakDancer^44^ v.1.1.2 and Pindel^45^ v.0.2.5b9. First, BreakDancer was used to detect structural variations with parameter “-q 20 -y 20 -r 1”. Secondly, the output of BreakDancer was used as an input for Pindel using the parameter “-x 4 -breakdancer” to increase the sensitivity and specificity. To merge the results from BreakDancer and Pindel, two structural variants with a distance between the two breakpoints of less than 100 bp were considered the same structural variation and merged. Owing to the inability of Pindel to detect intra- and inter-chromosomal translocations, only BreakDancer was used for their analysis. Furthermore, a structural variation was considered if it was present in at least 5% of the individuals in a given population.

For CNVs, we first generated a GC-content profile using gccount (http://bioinfo-out.curie.fr/projects/freec/src/gccount.tar.gz) with parameter “window = 1000 step = 1000” to normalize non-uniform read coverage of genomic position. Then, Control-FREEC^46^ v.11.0 was used to detect CNVs in 1-kb non-overlapping windows (bins) with parameter “ploidy = 2 window = 1000 step = 1000 mateOrientation=FR” for each high-depth individual (sequencing depth > 10X). Next, the sample-level copy numbers were combined to produce a matrix of copy numbers for each bin at the cohort level. To further reduce false positives, we filtered out the bins with a CNV rate of less than 1%. The affected genes were identified by the presence of overlapping regions with CNVs.

### Divergence and phylogenetic relationship

For divergence time estimation, 195 wild species accessions were assembled individually using MEGAHIT^40^ v.1.2.9 with default parameters. Then, the ‘fabales’ genes were downloaded from the BUSCO^17^ database (odb10), which contains 5,366 single-copy orthologues to predict the genes for 195 wild species accessions, CDC Frontier genome^11^ and *M. truncatula* genome^18^ (as outgroup) using GeneWise^47^ v.2.4.1 with the parameters “-both -sum -genesf”. On the basis of the gene annotations of 195 wild species accessions, only one sample with the longest average coding sequence (CDS) length was chosen for each wild species. The CDS sequences of single-copy genes in seven wild species, CDC Frontier and *M. truncatula* were extracted. For each single-copy family, multiple sequence alignment was performed using MUSCLE^48^ v.3.8.31 with default parameters and poorly aligned and divergent regions were eliminated using Gblocks^49^ v.0.91b with the parameter “-t=c”. The aligned matrix from each single-copy family was combined to construct the super aligned matrix. The maximum likelihood tree was constructed using RAxML^50^ v.8.2.12 with parameters “-f a -x 12345 -p 12345 -# 1000 -m GTRCATX”. Finally, divergence time was estimated by MCMCTree^51^ v.4.4 with three time-calibration points (0.007–0.013 Ma for *C. reticulatum*–*C. arietinum*, 12.2–17.4 Ma for *C. arietinum*–*C. pinnatifidum*, and 30.0–54.0 Ma for *C. arietinum–M. truncatula*) from the literature^52–54^.

To assess the relatedness among 195 wild accessions and 3,171 cultivated lines, the genetic distance matrix based on identity by state (IBS) was calculated through PLINK v1.90 with the parameter “--distance 1-ibs” using LD-pruned SNPs (--indep-pairwise 50 10 0.2) present on pseudomolecules. On the basis of the distance matrix, neighbour-joining phylogenetic trees were then constructed using ‘neighbor’ in PHYLIP^55^ v.3.6.

A PCA was undertaken to study the relatedness and clustering among cultivated chickpea accessions. The top 20 principal components (PCs) of the variance-standardized relationship matrix were estimated using EIGENSOFT^56^ v.7.2.0 with default parameters on LD-pruned SNPs present on pseudomolecules. PCA results were plotted using the R package ‘rworldmap’ (ref. ^57^).

### Diversity and genetic bottleneck

To characterize variation among populations, population differentiation statistics (*F*_ST_) were calculated in a 10-kb/2-kb sliding window using VCFtools v.0.1.13. A range of pairwise *F*_ST_ was calculated in the same combinations as for the ROD calculations. Tajima’s *D* was calculated using VCFtools (“--TajimaD 100000”) in 100-kb non-overlapping windows. A window was considered a selection window in the upper 90% of the population’s empirical distribution for ROD and *F*_ST_ statistics, along with a negative Tajima’s *D* value (less than −2). Genes located on the selection windows were identified, and functional enrichment of the KEGG pathway (v.87.0) and GO term for these candidate genes was conducted using the Fisher’s exact test with false discovery rate correction using EnrichmentPipeline^58^ (https://sourceforge.net/projects/enrichmentpipeline/).

For determining population size histories and split times, the SMC++ programme^59^ v.1.13.1 was used. Individuals with more than 20% missing data were filtered out. We built 20 random datasets of 150 genotypes. For each of the 20 datasets, SMC++ was used with a generation time of one year and a mutation rate of 6.5 × 10^−9^ (ref. ^60^). To avoid potential bias in the estimates owing to the long run of homozygosity, we filtered out homozygous regions longer than 5 kb in the 150 samples. For each of the 20 estimations, we used 5 different combinations of distinguished lineages, as suggested previously^59^. We then calculated the median of the 20 independent estimates for each time point.

SweeD (v.3.3.1) analysis was performed as previously^61^ on chromosomes Ca1 to Ca8. To keep calculation time and resource into reasonable burdens while staying conservative in pointing genomic regions as being likely to be under positive selection, 2 random sub-samples of 251 landraces, proportional to 2,439 landraces for each geographical region, were considered. The analysis computes in each sub-sample a CLR for each SNP along the genome. We used a grid value of 10,000 for each chromosome, corresponding roughly to computing a CLR ratio every 9 kb. We considered the highest 1% CLR values for each sample and kept them as candidate SNPs for positive selection of the positions detected in both samples. Owing to linkage disequilibrium, a high CLR value detected on an SNP can result from selection acting on a nearby gene. Therefore, we computed a list of intervals that are likely to be targeted by selection from the list of SNPs detected under selection, without pointing to particular SNPs but including all SNPs within 10 kb of each other.

Effect of nucleotide variations on protein function was predicted with SIFT 4G^21^ v.2.0.0. Putative deleterious mutations were identified with a SIFT score of less than 0.05. The *Medicago* genome was used as an outgroup to identify the derived alleles in the chickpea genome. Mutation burden was computed by counting the number of derived deleterious alleles present in constrained regions of the genome in each genotype as described before^8^.

### Genome-wide association analysis

Genome-wide association study (GWAS) analysis was performed using 3.94 million genome-wide SNPs and phenotypic data generated on 16 traits for 2 seasons and 6 locations. Only biallelic SNPs in cultivated genotypes were used in the GWAS analysis. Furthermore, the filtration was done with a minor allele frequency (MAF) cut-off of 0.05, missing rate cut-off of 0.8 and heterozygosity rate of 0.1. Marker trait association (MTA) analysis was then performed using a mixed linear model with the filtered HapMap file and phenotyping data. The first three PCs were used to control the population structure. The Manhattan plots and QQ plots were generated from the GWAS results. A *P* value of 3.16 × 10^−7^ was used to consider the MTA as significant.

### Identification of superior haplotypes

For haplotype analysis, we retained a SNP set for 3,171 cultivated chickpea lines according to the following criteria: (i) MAF > 0.001; and (ii) proportion of missing calls per SNP < 30%. The haplotypes present within trait-associated genes were examined and only homozygous calls were considered for haplotype analysis. The identified haplotypes were visualized in Flapjack^62^ v.1.19.09.04.

For the haplo–pheno analysis, haplotypes carrying only one genotype were removed from the analysis. The accessions were categorized on the basis of haplotype groups, and together with phenotypic data, superior haplotypes were identified^63^. Haplotype-wise means for 100SW, days to flowering (DF) and YPP were compared to define superior haplotypes. Duncan’s multiple range test was used for statistical significance.

### OCS approach

We used GEBV from the genomic prediction section for key production traits (YPP, 100SW, DF and days to maturity (DM)) to generate a genomic relationship matrix based on 754,576 SNPs. We used the breeding program implementation platform MateSel v.6.3 (http://matesel.une.edu.au) to generate an optimized mating design within desi, kabuli and intermediate types. The relative emphasis on the mean index versus co-ancestry was set by choosing the target degrees on the response surface^24^. We chose a target of 60 degrees to minimize the increase in population co-ancestry (maximize population genetic diversity) while achieving an acceptable rate of genetic gain. As this study aimed to maintain a diverse pre-breeding pool while making economic improvements, we followed the conservative approach for ‘evolving gene banks’ (ref. ^23^).

We generated unique economic indices for desi and kabuli chickpea, which were calculated on a US$ per ha basis and included yield (average GEBV for YPP over 9 sites) with a bonus price for large seeds (when average GEBV for 100SW over 9 sites exceeded the average for kabuli of +5.9 g) and earliness (average GEBV for DF and DM over 9 sites < 0 days). The base price for chickpea was assumed to be US$400 per tonne, and YPP was converted to an equivalent grain yield value per hectare by assuming that the mean YPP of 18 g per plant is equivalent to 1.8 tonnes per hectare. The index was also adjusted for a price bonus for large seeds and earliness as follows. The starting values for GEBV for 100SW are low in desi candidates (mean −4.0 g) and high in kabuli candidates (mean +5.9 g). Hence, the starting value for a price bonus for 100SW begins at GEBV + 5.9 g, and there is no bonus below this value. The price bonus per gram (GEBV 100SW > 5.9 g) is US$35 per gram, which is added to the base price. Similarly, a bonus was provided in price per tonne for GEBV earliness (average of GEBV DF and GEBV DM). The average GEBV earliness in the desi group was −1.6 days, and in the kabuli group was +2.4 days. The starting value for a price bonus for earliness begins at average GEBV 0 days; there is a bonus for negative values of US$10 per day added to the base price and no bonus for positive values.

### Genomic prediction analyses

#### Prediction using the interaction of genomic and environmental covariates

As described previously^25^, three models, a basic model (E + L) with main effects of environments (E) and lines (L), a model (E + L + G) including the main effects of markers, and a genomic by environment interaction model (E + L + G + GE) were used. Three different SNP datasets (G1, cultivated accessions; G2, wild accessions; and G3, G1 + G2) were used as a genomic matrix (G), post-conventional quality controls on missing values (<20%) and MAF (>0.05). Phenotyping data for nine traits across 12 different year × location combinations were used. The Pearson’s correlation coefficient between observed phenotype and predicted genomic breeding value was used to estimate the accuracy of genomic prediction. Three different random cross-validation (CV) schemes, CV1 (evaluate the prediction accuracy of models when a certain percentage of lines are not observed in any environment), CV2 (estimates the prediction accuracy of models when some lines are evaluated in some environments but not in others) and CV0 (predicts an unobserved environment using the remaining environments as a training set) were used. CV1 and CV2 with fivefold cross-validation were implemented to generate the training and testing sets, and the prediction accuracy was assessed for each testing set. The permutation of the five subsets led to five possible training and validation datasets. This procedure was repeated 20 times, and 100 runs were performed for each trait–environment combination on each population. The same partition was used for the analysis of all the GS models. For CV0, each environment was predicted using the remaining environments. For fitting the GS models, the R package Bayesian Generalized Linear Regression (BGLR)^64^ v.1.0.7 was used.

#### Prediction using the WhoGEM method

For WhoGEM analysis, 1,318 accessions with the validated geographical location were selected and used as a reference dataset. The SNP dataset was filtered for missing (>0.1) and MAF (<0.01) and used for a detailed search with ADMIXTURE^65^ v.1.3.0 between *K* = 19 and *K* = 30 to identify the most likely number of admixture components. To confirm the admixture value, another method, DAPC (discriminant analysis of principal components), was used. The optimal number of admixture components in the WhoGEM method was obtained by comparing the predicted and recorded locations (ProvenancePredictor algorithm^26^) and fixed to *K* = 23.

A general linear model explored the relationships between the phenotypes and admixture components, and land types. A forward–backward algorithm was used to reduce the set of predictors to the most significant ones. The model is fitted on the whole dataset, and the significant factors are identified and conserved. A negative control (a model without any genetics (called environment-only)) is also fitted to the data. The models were fitted on the whole dataset, and the significant factors were identified and conserved.

A test of WhoGEM significance is given by a likelihood ratio test comparing the WhoGEM-based model and the environment-only-based model. The performances of the three models (full WhoGEM-based model, additive and environment-only model) are then evaluated using 100–300 replicates of a fivefold cross-validation scheme.

### Prediction using a haplotype-based approach

The SNP set was filtered, first by excluding all markers with more than two called alleles, missing (>10%) and MAF (<5%). A subset of 124,833 (20%) of 2.4 million high-quality SNPs were randomly selected to reduce the computational load in further analyses. Those SNPs were used to construct LD blocks and estimate local GEBVs for haplotypes of those LD blocks. Details on the method used to calculate local GEBVs for haplotypes of LD blocks are described in a previous report^27^.

We also ran a ridge-regression best linear unbiased prediction (BLUP) model in the R-package rrBLUP (ref.^66^) v.4.6.0 to predict marker effects for seven agronomic traits, then summed up the predicted allelic effects of each observed haplotype for all genome-wide LD blocks. Finally, we estimated variances among local GEBVs for haplotypes within each LD block to highlight regions in the genome showing molecular variation linked to observed phenotypic variation for the agronomic traits measured in the field trials.

### Reporting summary

Further information on research design is available in the Nature Research Reporting Summary linked to this paper.

## Online content

Any methods, additional references, Nature Research reporting summaries, source data, extended data, supplementary information, acknowledgements, peer review information; details of author contributions and competing interests; and statements of data and code availability are available at 10.1038/s41586-021-04066-1.

### Supplementary information

Supplementary Information This file contains Supplementary Notes and References – see contents page for details.

Reporting Summary

Supplementary Data 1

Supplementary Data 2

Supplementary Data 3

Supplementary Data 4

Supplementary Data 5

Supplementary Data 6

Supplementary Data 7

Supplementary Data 8

Supplementary Data 9

Supplementary Data 10

### Source data

Source Data Fig. 1

Source Data Fig. 3

Source Data Extended Data Fig. 8

Source Data Extended Data Fig. 11

Source Data Extended Data Fig. 12

## Figures and Tables

**Extended Data Table 1:**
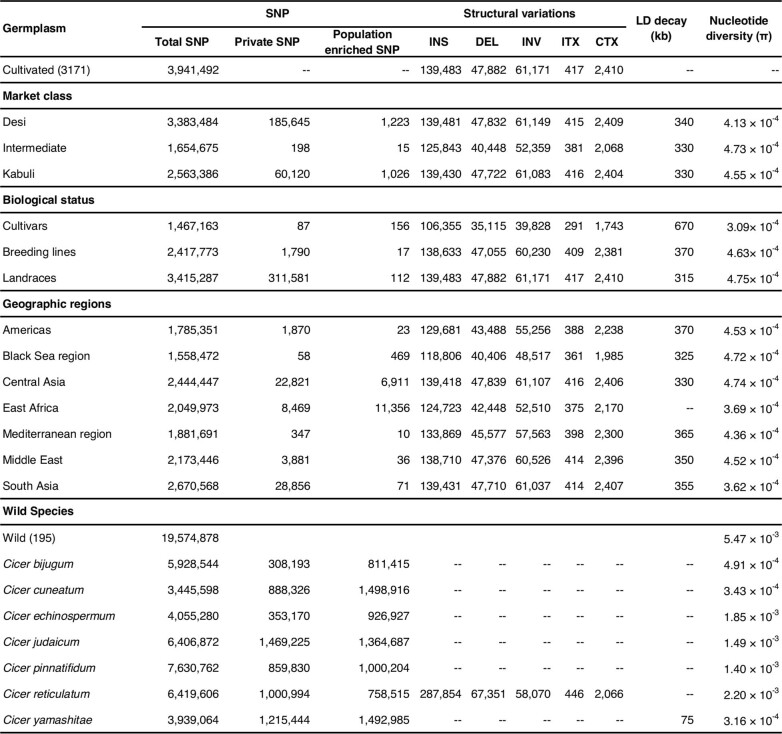
Summary of genome diversity features

## Data Availability

The data that support the findings of this study have been deposited in the NCBI under accession code BioProject: PRJNA657888. The chickpea pan-genome assembly and annotations developed in this study are available at 10.6084/m9.figshare.16592819. The variant calls for each accession and phenotype data are available to download at https://cegresources.icrisat.org/cicerseq. Manhattan and QQ-plots for GWAS analysis are available at 10.6084/m9.figshare.15015309 and 10.6084/m9.figshare.15015315, respectively. Source data are provided with this paper.
