# Author Correction: A chickpea genetic variation map based on the sequencing of 3,366 genomes

**DOI:** 10.1038/s41586-022-04660-x

**Published:** 2022-03-25

**Authors:** Rajeev K. Varshney, Manish Roorkiwal, Shuai Sun, Prasad Bajaj, Annapurna Chitikineni, Mahendar Thudi, Narendra P. Singh, Xiao Du, Hari D. Upadhyaya, Aamir W. Khan, Yue Wang, Vanika Garg, Guangyi Fan, Wallace A. Cowling, José Crossa, Laurent Gentzbittel, Kai Peter Voss-Fels, Vinod Kumar Valluri, Pallavi Sinha, Vikas K. Singh, Cécile Ben, Abhishek Rathore, Ramu Punna, Muneendra K. Singh, Bunyamin Tar’an, Chellapilla Bharadwaj, Mohammad Yasin, Motisagar S. Pithia, Servejeet Singh, Khela Ram Soren, Himabindu Kudapa, Diego Jarquín, Philippe Cubry, Lee T. Hickey, Girish Prasad Dixit, Anne-Céline Thuillet, Aladdin Hamwieh, Shiv Kumar, Amit A. Deokar, Sushil K. Chaturvedi, Aleena Francis, Réka Howard, Debasis Chattopadhyay, David Edwards, Eric Lyons, Yves Vigouroux, Ben J. Hayes, Eric von Wettberg, Swapan K. Datta, Huanming Yang, Henry T. Nguyen, Jian Wang, Kadambot H. M. Siddique, Trilochan Mohapatra, Jeffrey L. Bennetzen, Xun Xu, Xin Liu

**Affiliations:** 1 Center of Excellence in Genomics and Systems Biology, International Crops Research Institute for the Semi-Arid Tropics (ICRISAT), Hyderabad, India; 2 State Agricultural Biotechnology Centre, Centre for Crop and Food Innovation, Murdoch University, Murdoch, Western Australia Australia; 3 BGI-Qingdao, BGI-Shenzhen, Qingdao, China; 4 China National GeneBank, BGI-Shenzhen, Shenzhen, China; 5 College of Life Sciences, University of Chinese Academy of Sciences, Beijing, China; 6 Institute of Crop Germplasm Resources, Shandong Academy of Agricultural Sciences (SAAS), Jinan, China; 7 ICAR–Indian Institute of Pulses Research, Kanpur, India; 8 Genebank, ICRISAT, Hyderabad, India; 9 University of Georgia, Athens, GA USA; 10 BGI-Shenzhen, Shenzhen, China; 11 State Key Laboratory of Agricultural Genomics, BGI-Shenzhen, Shenzhen, China; 12 The UWA Institute of Agriculture, and School of Agriculture and Environment, The University of Western Australia, Perth, Western Australia Australia; 13 Biometrics and Statistics Unit, International Maize and Wheat Improvement Center (CIMMYT), Texcoco, Mexico; 14 Digital Agriculture Laboratory, Skolkovo Institute of Science and Technology, Moscow, Russia; 15 Queensland Alliance for Agriculture and Food Innovation, The University of Queensland, St Lucia, Queensland Australia; 16 International Rice Research Institute (IRRI), South-Asia Hub, ICRISAT, Hyderabad, India; 17 Laboratoire Ecologie Fonctionnelle et Environnement, Université de Toulouse, CNRS, Toulouse, France; 18 Institute for Genomic Diversity, Cornell University, Ithaca, NY USA; 19 Department of Plant Sciences, University of Saskatchewan, Saskatoon, Saskatchewan Canada; 20 ICAR–Indian Agricultural Research Institute (IARI), New Delhi, India; 21 Rajmata Vijayaraje Scindia Krishi Vishwa Vidyalaya, Gwalior, India; 22 Junagadh Agricultural University, Junagadh, India; 23 Rajasthan Agricultural Research Institute (RARI), Durgapura, India; 24 Department of Agronomy and Horticulture, University of Nebraska–Lincoln, Lincoln, NE USA; 25 DIADE (Diversity-Adaptation-Development of Plants), Université de Montpellier, Institut de Recherche pour le Développement (IRD), Montpellier, France; 26 International Centre for Agricultural Research in the Dry Areas (ICARDA), Cairo, Egypt; 27 International Centre for Agricultural Research in the Dry Areas (ICARDA), Rabat, Morocco; 28 Rani Lakshmi Bai Central Agricultural University, Jhansi, India; 29 National Institute of Plant Genome Research, New Delhi, India; 30 Department of Statistics, University of Nebraska–Lincoln, Lincoln, NE USA; 31 School of Plant Sciences, University of Arizona, Tucson, AZ USA; 32 Department of Plant and Soil Science, University of Vermont, Burlington, VT USA; 33 University of Calcutta, Kolkata, India; 34 Guangdong Provincial Academician Workstation of BGI Synthetic Genomics, BGI-Shenzhen, Shenzhen, China; 35 Division of Plant Sciences, University of Missouri, Columbia, MO USA; 36 James D. Watson Institute of Genome Science, Hangzhou, China; 37 Indian Council of Agricultural Research (ICAR), New Delhi, India; 38 Department of Genetics, University of Georgia, Athens, USA; 39 Guangdong Provincial Key Laboratory of Genome Read and Write, BGI-Shenzhen, Shenzhen, China; 40 BGI-Beijing, BGI-Shenzhen, Beijing, China; 41 BGI-Fuyang, BGI-Shenzhen, Fuyang, China

**Keywords:** Structural variation, Agricultural genetics, Plant breeding, Natural variation in plants, Plant breeding

Correction to: *Nature* 10.1038/s41586-021-04066-1 Published online 10 November 2021

In Extended Data Fig. 1 of this Article, the labels ‘Market class’ and ‘Biological status’ were inadvertently swapped. In the corresponding figure legend, “Track 1: Biological status; Track 2: Market class;” should have been “Track 1: Market class; Track 2: Biological status;”. The original Article has been corrected online

