# Supplementary material for: A chickpea genetic variation map based on the sequencing of 3,366 genomes
Source: Nature. 2021 Nov 10;599(7886):622–7. doi: 10.1038/s41586-021-04066-1 (PMC8612933; doi:10.1038/s41586-021-04066-1)
Supplement: Supplementary file 1 — This file contains Supplementary Notes and References – see contents page for details. [file 41586_2021_4066_MOESM1_ESM.pdf]

nature portfolio

https://doi.org/10.1038/s41586-021-04066-1

Supplementary information

A chickpea genetic variation map based on the sequencing of 3,366 genomes

In the format provided by the authors and unedited

Supplementary Notes

A chickpea genetic variation map based on the sequencing of 3,366 genomes

Rajeev K Varshney[1,2*], Manish Roorkiwal[1], Shuai Sun[3,4], Prasad Bajaj[1], Annapurna Chitikineni[1], Mahendar Thudi[1,5], Narendra P Singh[6], Xiao Du[3,4], Hari D Upadhyaya[7,8], Aamir W Khan[1], Yue Wang[3,4], Vanika Garg[1], Guangyi Fan[3,4,9,10], Wallace A. Cowling[11], Jose Crossa[12], Laurent Gentzbittel[13], Kai Peter Voss-Fels[14], Vinod Kumar Valluri[1], Pallavi Sinha[1,15],Vikas K Singh[1,15], Cécile Ben[13,16], Abhishek Rathore[1], Ramu Punna[17], Muneendra K Singh[1], Bunyamin Tar'an[18], Chellapilla Bharadwaj[19], Mohammad Yasin[20], Motisagar S. Pithia[21], Servejeet Singh[22], Khela Ram Soren[6], Himabindu Kudapa[1], Diego Jarquin[23], Philippe Cubry[24], Lee T Hickey[14], Girish Prasad Dixit[6], Anne-Céline Thuillet[24], Aladdin Hamwieh[25], Shiv Kumar[26], Amit A. Deokar[18], Sushil K Chaturvedi[27], Aleena Francis[28], Reka Howard[29], Debasis Chattopadhyay[28], David Edwards[11], Eric Lyons[30], Yves Vigouroux[24], Ben J. Hayes[14], Eric von Wettberg[31], Swapan K Datta[32], Huanming Yang[10,33], Henry T. Nguyen[34], Jian Wang[10,35], Kadambot H.M. Siddique[11], Trilochan Mohapatra[36], Jeffrey L. Bennetzen[37], Xun Xu[9,38], Xin Liu[9,10,39,40,*]

[1]Center of Excellence in Genomics & Systems Biology, International Crops Research Institute for the Semi-Arid Tropics (ICRISAT), Hyderabad, India
[2]State Agricultural Biotechnology Centre, Centre for Crop and Food Innovation, Murdoch University, Murdoch, Western Australia, Australia
[3]BGI-Qingdao, BGI-Shenzhen, Qingdao, China
[4]China National GeneBank, BGI-Shenzhen, Shenzhen, China
[5]Institute of Crop Germplasm Resources, Shandong Academy of Agricultural Sciences (SAAS), Jinan, China
[6]ICAR-Indian Institute of Pulses Research, Kanpur, India
[7]Genebank, International Crops Research Institute for the Semi-Arid Tropics (ICRISAT), Hyderabad, India
[8]University of Georgia, Athens, GA, USA
[9]BGI-Shenzhen, Shenzhen, China
[10]State Key Laboratory of Agricultural Genomics, BGI-Shenzhen, Shenzhen, China
[11]The UWA Institute of Agriculture, and School of Agriculture and Environment, The University of Western Australia, Perth, WA, Australia
[12]Biometrics and Statistics Unit, International Maize and Wheat Improvement Center, (CIMMYT), Mexico and Colegio de Postgraduados, Mexico
[13]Digital Agriculture Laboratory, Skolkovo Institute of Science and Technology, Moscow, Russia
[14]Queensland Alliance for Agriculture and Food Innovation, The University of Queensland, St Lucia, QLD, Australia
[15]International Rice Research Institute (IRRI), South-Asia Hub, ICRISAT, Hyderabad, India
[16]Laboratoire Ecologie Fonctionnelle et Environnement, Université de Toulouse, CNRS, Toulouse, France
[17]Institute for Genomic Diversity, Cornell University, Ithaca, New York, USA
[18]Department of Plant Sciences, University of Saskatchewan, Saskatoon, SK, Canada
[19]ICAR–Indian Agricultural Research Institute (IARI), New Delhi, India
[20]Rajmata Vijayaraje Scindia Krishi Vishwa Vidyalaya, Gwalior, India
[21]Junagadh Agricultural University, Junagadh, India
[22]Rajasthan Agricultural Research Institute (RARI), Durgapura, India
[23]Department of Agronomy and Horticulture, University of Nebraska-Lincoln, Lincoln, NE, USA
[24]DIADE (Diversity-Adaptation-Development of plants), Université de Montpellier, IRD (Institut de Recherche pour le Développement), Montpellier, France
[25]International Centre for Agricultural Research in the Dry Areas (ICARDA), Cairo, Egypt
[26]International Centre for Agricultural Research in the Dry Areas (ICARDA), Rabat, Morocco
[27]Rani Lakshmi Bai Central Agricultural University, Jhansi, India
[28]National Institute of Plant Genome Research, New Delhi, India

[29]Department of Statistics, University of Nebraska-Lincoln, Lincoln, NE, USA
[30]School of Plant Sciences, University of Arizona, Tucson, AZ, USA
[31]Department of Plant and Soil Science, University of Vermont, Burlington, VT, USA
[32]University of Calcutta/ VISVA-BHARATI, Santiniketan, West Bengal
[33]Guangdong Provincial Academician Workstation of BGI Synthetic Genomics, BGI-Shenzhen, Shenzhen, China
[34]Division of Plant Sciences, University of Missouri, Columbia, MO, USA
[35]James D. Watson Institute of Genome Science, Hangzhou, China
[36]Indian Council of Agricultural Research (ICAR), New Delhi, India
[37]Department of Genetics, University of Georgia, Athens, USA
[38]Guangdong Provincial Key Laboratory of Genome Read and Write, BGI-Shenzhen, Shenzhen, China
[39]BGI-Beijing, BGI-Shenzhen, Beijing, China
[40]BGI-Fuyang, BGI-Shenzhen, Fuyang, China

*Correspondence should be addressed to: r.k.varshney@cgiar.org / rajeev.varshney@murdoch.edu.au; and liuxin@genomics.cn

Table of Contents

Whole genome sequencing and genome wide variations 2

Linkage disequilibrium (LD) decay 3

Private and population enriched alleles 3

Pan-genome and presence-absence variations 4

Structural variations 5

Species divergence 6

Phylogenetic analysis of wild accessions 7

Post-domestication divergence 7

Breeding bottlenecks 8

Genomic regions undergone selection 9

Genetic loads 9

Genome wide association studies 11

Haplotype analysis 12

Optimal contribution selection (OCS) 13

Genomic prediction approaches 14

- Estimating GEBVs by consideration of interaction of genomic and environmental covariates 14
- Prediction of trait performance by implementing genotype and geographical coordinates using the WhoGEM 15
- Haplotype based local genomic estimated breeding values (local GEBVs) 16

Whole genome sequencing and genome wide variations

Whole genome sequencing data on 3,366 chickpea germplasm lines, including 3,171 cultivated and 195 wild accessions, representing seven annual wild *Cicer* species from all three major germplasm categories ("genepools"): the primary genepool (GP1) *C. echinospermum* (9), and *C. reticulatum* (28); the secondary genepool (GP2), *C. bijugum* (41), *C. judaicum* (68), *and C. pinnatifidum* (39); and the tertiary genepool (GP3), *C. cuneatum* (4) and *C. yamashitae* (6) (Supplementary Data 1 Table 1). The dataset includes 396 (12.5%) breeding lines, 152 (4.8%) cultivars, 2,439 (76.9%) landraces, and 184 (5.8%) of accessions with unknown utilization status. The cultivated accessions included 1,782 (56.2%) desi, 1,266 (39.9%) kabuli, 113 (3.6%) intermediate and 10 (0.3%) accessions with unknown market class. All these cultivated accessions were categorized into one of seven populations based on geographical regions: Americas (208; 6.56%), Black Sea region (86; 2.71%), Central Asia (717; 22.61%), East Africa (154; 4.86%), Mediterranean region (235; 7.41%), Middle East (475; 14.98%) and South Asia (1,235; 38.95%). A total of 61 (1.92%) of the accessions had unknown geographic origins.

Alignment of 21.33 tera-base pairs (Tbp) of WGS data (including 1.75 Tbp from ref.[2]) to the CDC Frontier reference genome[11], resulted in an average mapping of 80.68% of the genome per accession (38.07–83.40% for wild; 26.22–83.88% for cultivated; Supplementary Data 1 Table 2). We identified 3.94 million SNPs in 3,171 cultivated germplasm lines, ranging from 1.69–3.92 million (average 3.61 million) SNPs per accession relative to the CDC Frontier reference genome (Supplementary Data 1 Table 3 and 4). Similarly, we identified 19.57 million SNPs in 195 wild accessions ranging from 7.96–19.36 million (average 13.72 million) (Supplementary Data 1 Table 5 and 6). Many SNPs were on scaffolds, both in cultivated (1.47 million SNPs; Supplementary Data 1 Table 3 and 4) and wild accessions (3.91 million SNPs; Extended Data Table 1; Supplementary Data 1 Table 5 and 6). SNP density was highest on the Ca4 pseudomolecule in cultivated and the Ca8 pseudomolecule in wild accessions. The average non-synonymous to synonymous ratio of 1.53 in cultivated accessions indicates positive selection, while 0.94 in the wild accessions may indicate a neutral or slightly negative selection (Supplementary Data 1 Table 7). We observed 2,974 SNPs leading to stop loss (1,455) and start loss (1,519) variant effects in wild accessions and 4,118 SNPs leading to stop gain variant effects in cultivated accessions (Supplementary Data 1 Table 7).

Linkage disequilibrium (LD) decay

In terms of linkage disequilibrium (LD) decay, we observed less LD in landraces (315 kb), compared to breeding lines (370 kb) and cultivars (670 kb) (Supplementary Data 2 Table 1; Extended Data Fig. 2a). Under directional positive selection, nucleotide diversity and haplotype diversity decrease while LD increases[67]. LD decay in the different market classes did not differ significantly in this study compared to our earlier study (ref.[2]; Supplementary Data 2 Table 1; Extended Data Fig. 2b). Our analysis showed slower LD decay in the East Africa region (Supplementary Data 2 Table 1; Extended Data Fig. 2c). Similarly, we observed shorter LD decay in germplasm lines from Turkey, Iran and Syria (Supplementary Data 2 Table 2; Extended Data Fig. 2d).

Private and population enriched alleles

We identified private SNPs (alleles present in ≥ 4 accessions within a population and absent in other populations) and population-enriched SNPs (≥ 20% present in one population and ≤ 2% in others) classified by geographical region (Supplementary Data 3 Table 1) and biological status (Supplementary Data 3 Table 2). The genetic variants shared by any two populations are likely to represent standing variation inherited from their most recent common ancestral population. In contrast, new mutations observed in any population might have arisen due to new recombination or gene flow from the other population growing next to that population in recent times. In our analysis, the South Asia population harboured the largest number of private SNPs (28,856), followed by Central Asia (22,821). East African accessions harboured the highest number of population-enriched SNPs (11,356), followed by Central Asia (6,911) (Supplementary Data 3 Table 1). From a biological status perspective, the fewest population-enriched SNPs were found among breeding lines, with none being found on pseudomolecules Ca1, Ca7 and Ca8 (Supplementary Data 3 Table 2).

Among cultivated accessions, desi (small and darker seed) genotypes possessed a larger number of private (185,645) as well as population-enriched (1,223) SNPs than kabuli (lighter coloured and larger seeds) (60,120 private SNPs; 1,026 population-enriched SNPs) or intermediate (dark or light coloured, small or medium sized and round) (198 private SNPs; 15 population-enriched SNPs) genotypes (Supplementary Data 3 Table 3). Among wild species accessions, *C. judaicum* had the highest number of private SNPs (1,469,225), and *C. cuneatum* had the most population-enriched SNPs (1,498,916) (Supplementary Data 3 Table 4). *C.*

bijugum and *C. reticulatum* had the fewest private SNPs (308,193) and population-enriched SNPs (758,515), respectively.

Pan-genome and presence-absence variations

A typical pan-genome consist of core genome (shared among all individuals of a species) and dispensable genome (present in some individuals but not all) representing entire genetic variation and architecture of any species. A pan-genome can further be characterized as closed (where the size does not further increase after a certain number of individuals are added) and open (size tends to increase with the addition of each individual). A chickpea pan-genome was developed using the reference genome of CDC Frontier[11] (as foundation genome), together with ICC 4958[12,37] (a desi accession), PI 489777[13] (a *C. reticulatum* accession) and *de novo* assembled sequences from 3,171 cultivated and 28 *C. reticulatum* accessions (Supplementary Data 4 Table 1). We used an iterative mapping and assembly approach for pangenome construction, since this is the most appropriate for the short reads sequencing data. However, the traditional representation of linear reference genomes limits all the alleles present across different genotypes in a population at a locus. Although graph-based pangenome representation has an advantage over linear pangenome representation, our approach seems appropriate in the absence of draft genomes for all wild species accessions used in the study. To predict genes models, *ab initio* and homology-based prediction approaches were employed. For the homology-based approach, protein sequences from soybean (*Glycine max*)[68], pigeon pea (*Cajanus cajan*)[69], Medicago (*Medicago truncatula*)[18], Arabidopsis (*Arabidopsis thaliana*)[70] and the SWISS-PROT[71] (release-2018_07) plant protein database, were used to predict gene models through GeneWise[47] v2.4.1. Further, AUGUSTUS[72] v3.1 was then used for *ab initio* annotation with default parameters, with the Arabidopsis data set as a training set. EVM[73] v1.1.1 was used to integrate the results from both *ab initio* and homology-based approaches with prediction weights of 1 and 10 separately. The genes were annotated by aligning the protein sequences translated from gene models to the KEGG v87.0 and NCBI NR databases through BLASTP[43] with the threshold of E-value ≤1e-5 and the rank of best alignment ≤5. Finally, genes with NR and KEGG annotations and homologous sequences with coverage ≥40 were retained (Supplementary Data 4 Table 2).

We further identified the potentially new genes compared to the existing CDC Frontier genes, using blastp from BLAST v2.2.31. Genes with coverage < 80% or identity < 80% were considered potential new genes. Further, we aligned the additional genes to the CDC Frontier

genes for alignment identity and coverage. We found that only 38.40% of these additional genes can be aligned to genes in the reference genome (E-value <1e-5). Among those aligned genes, the average identity of 44.14% and an average coverage of 60.81%, reflecting low similarity among the additional genes and the genes in the reference genome. Only a small proportion of genes were well aligned (≥80% identity and ≥80% coverage) to the reference. In the updated pan-genome, we have observed 1,601 additional genes, of which 1,582 are potentially novel. Furthermore, based on enrichment analysis, these genes are enriched in metabolism and biosynthesis of secondary metabolites, indicating their possible roles in different groups to result in different efficiencies of metabolism, adaptation, or resistance to disease and pest. Modelling analysis of the pan- and core-genome depicted an increase and decrease in the number of genes with each added genotype, respectively (Fig. 1a). To detect gene presence/absence variations (gene PAVs) for the 2,258 cultivated accessions with sequence depth >10, a strategy based on reads coverage was used (Supplementary Data 4 Table 4). The reads of each sample were aligned to the pan-genome using BWA[31] v0.7.15 with default parameters, and then the coverage for each gene was calculated using the SAMTools v1.2 depth command. Coverage of 0.3 for gene region was used as the threshold to distinguish the PAV genes, and a VCF-format file for each sample was generated. Then, the results of gene PAVs of each sample were merged with BCFTools[74] v1.4 with the parameter "bcftools merge".

We further analysed the effect of PAVs on protein-coding genes. Of the 1,867 PAV genes, three (Ca_04548, Ca_04560 and Ca_04561) were found in the previously identified *QTL-hotspot* region[75]. These genes encode for transcription initiation factor TFIID subunit-like protein (Ca_04548), VAN3-binding protein-like isoform X2 (Ca_04560) and zinc finger C3HC4 type protein (Ca_04561).

Structural variations

We analyzed all cultivated and *C. reticulatum* accessions with >10X coverage in reference to the CDC Frontier genome with BreakDancer[44] and Pindel[45] to identify various types of structural variations (SV) like insertions (INS), deletions (DEL), inversions (INV), intra-chromosomal translocations (ITX) and inter-chromosomal translocations (CTX). To avoid inconsistency and imprecision of the SV breakpoints in sample sets, a threshold of 1 kb distance apart of breakpoints was used to merge the SV from all samples. To reduce the false-positive SV discovery from NGS short reads, only an SV present in at least six individuals (i.e. variant frequency more than 1%) was considered. We also identified genes affected by overlapping

regions with INS, DEL, INV, ITX and CTX breakpoints. For instance, in cultivated accessions, we identified 139,483 INS, 47,882 DEL, 61,171 INV, 417 ITX, and 2,410 CTX affecting 9,007, 4,760, 4,268, 60, and 289 genes, respectively (Extended Data Table 1, Supplementary Data 5 Table 1). Similarly, the *C. reticulatum* accessions had 287,854 INS, 67,351 DEL, 58,070 INVs, 446 ITX, and 2,066 CTXs affecting 18,223; 6,699; 4,654; 78 and 396 genes, respectively (Extended Data Table 1, Supplementary Data 5 Table 1). The greater number of SVs in the *C. reticulatum* accessions were expected because of the greater diversity in these materials and the fact that a cultivated chickpea genome was used as the reference.

Based on market class, kabuli accessions had fewer structural variations than desi accessions (Extended Data Table 1). Furthermore, all structural variations, except deletions, were less abundant in cultivars than breeding lines and landraces (Extended Data Table 1).

We also identified genomic regions harbouring SV clusters among cultivated accessions. A region containing more than two SVs of less than 500 bp adjacent distance was identified as one SV cluster. When more than 20% of all individuals had shared individual-level SV clusters, the region was identified as one clustering region. We identified 1,989 regions as SV clusters in cultivated chickpea, with the vast majority on Ca4 and the least on Ca8 (Supplementary Data 5 Table 2).

Based on the mapping depth of each base of the reference genome, we identified 793 gene gain copy number variations (CNV) and 209 gene loss CNV in cultivated accessions, and 643 gene gain and 247 gene loss CNV in *C. reticulatum* accessions. In cultivated accessions, these gene gain CNV spanned ~12.45 Mb (average of 15.69 kb), and the gene loss CNV spanned ~5.78 Mb (average length 27.63 kb). In the *C. reticulatum* accessions, the gene gain CNV spanned ~14.82 Mb (average length 23.05 kb) and the gene loss CNV spanned ~14.71 Mb (average length 59.57 kb). *C. reticulatum* accessions had 99 genes with both CNV gain and loss and 27,119 genes with normal copies (Supplementary Data 5 Table 3).

Species divergence

Of the nine annual *Cicer* species, *C. arietinum* L. is the only cultivated species. With an aim to understand the speciation and species divergence time in the eight species for which we generated data, the "fabales" genes were downloaded from the BUSCO[17] database (odb10) based on homolog-based gene annotation (including homolog searching and gene structure

prediction), which contains 5,366 single-copy orthologs to predict the genes for 195 wild species accessions, based on the CDC frontier genome and *M. truncatula*[18] genome (as outgroup). We used an MCMCtree analysis[51] considering three calibration points (0.007- 0.013 MYA for *C. reticulatum–C. arietinum*,12.2- 17.4 MYA for *Cicer arietinum – Cicer pinnatifidum*, and 30.0-54.0 MYA for *C. arietinum- M. truncatula*) for estimating species divergence time. We estimated divergence time as ~0.0126 MYA between *C. arietinum* and *C. reticulatum* ancestors. Their common ancestor had diverged ~15.3 (14-16.2) MYA from the *C. echinospermum* lineage. In addition, our analysis estimated that *C. judaicum* and *C. bijugum* had diverged by ~8.3 (3.2-12.7) MYA, and their common ancestor *C. pinnatifidum* had diverged ~13.3 (10.2-16.3) MYA. We have shown these species divergence time Extended Data Fig. 3a.

Phylogenetic analysis of wild accessions

We constructed a phylogenetic tree using all 195 wild accessions from seven *Cicer* species, grouped into six clusters (Clusters I- VI). We observed grouping all accessions of *C. judaicum*, *C. yamashitae* and *C. cuneatum* in Clusters III, V and VI, respectively (Extended Data Fig. 3b). Clusters III and IV were divided into two sub-clusters each. Sub-clusters IIIa and IIIb contain only *C. judaicum* accessions. Cluster IVa includes all *C. reticulatum* accessions and one *C. echinospermum* accession (ICC 20192; green colour), while Cluster IVb included all *C. echinospermum* accessions and one accession of *C. reticulatum* (ICC 73071; golden yellow colour). One accession of *C. pinnatifidum* (ICC 20168; red colour) was grouped with the *C. bijugum* accessions in Cluster II, and one accession of *C. bijugum* (ICC 20167; blue colour) was grouped with *C. pinnatifidum* accessions in Cluster I.

Post-domestication divergence

Effective seed dispersal (mostly by post-shattering in legumes) is crucial for achieving greater fitness in most wild species. Haplotypic differences were observed between wild *Cicer* species and cultivated chickpea in a homolog (*Ca_25684*) of SHATTERPROOF2/Agamous-like MADS-box protein (AGL5), with a transition/transversion from 'T' or 'A' (in 186/195 wild accessions) to 'C' (in 3,170/3,171 cultivated accessions) in the coding region (Supplementary Data 7). We found the "T" allele in all accessions of *C. bijugum*, *C. cuneatum*, *C. judaicum, C. yamashitae,* 30/39 accessions of *C. pinnatifidum,* 20/28 accessions of *C. reticulatum* and 8/9 accessions of *C. echinospermum.* The "A" allele was present exclusively in the remaining 9 *C.*

pinnatifidum species. The cultivated species specific allele ("C") was present in 8 accessions of *C. reticulatum* and "T/C" heterozygosity in 1 accession of *C. echinospermum* species. Only one cultivated genotype (ICC 16369) contained the "T" allele of wild species, suggesting that this genotype may have been misidentified as a cultivated accession. Similar mislabeling during collection expeditions, perhaps caused by morphological similarities in the groups of these species, has also been reported previously in the case of the PI593709 (ILWC 242) accession[76].

Breeding bottlenecks

By using inference methods to predict historical effective population size[59], chickpea history was found to be associated with a strong bottleneck beginning around 10,000 years ago, with the population size reaching its minimum ~1,000 years ago (Extended Data Fig. 6). Next, a very strong expansion of the population occurred, intensifying within the last 400 years. We observed the impact of these domestication and breeding bottlenecks on nucleotide diversity (π) in different groups. For instance, high nucleotide diversity was found in wild accessions (5.47×10^{-3}) relative to cultivated accessions. Among different populations of cultivated accessions, π ranged from 4.75×10^{-4} for landraces to 3.09×10^{-4} for cultivars. Breeding lines had higher nucleotide diversity (4.63×10^{-4}) than cultivars (3.09×10^{-4}) (Supplementary Data 6 Table 1).

To further understand the genetic relationships between cultivated and wild chickpea, we constructed neighbour-joining trees in PHYLIP[55] v3.6 using the SNPs on the pseudomolecules based on the biological status, market class and geographic origin. We observed a grouping of wild accessions as a separate cluster while the cultivated accessions formed three clusters (Extended Data Fig. 7). We found clustering of one cultivated accession (ICC 16369) from East Africa with wild accessions instead of the cultivated group.

Genomic regions undergone selection

Strong positive selection can also be detected by scanning the genome for deviation from expected SNP frequencies distribution or strong differentiation. In general, domestication, selection and adaptation have contributed to genome-wide divergence and stratification in chickpea populations. Three parameters (ROD, F_{ST} and Tajima's D) were used to identify genomic regions that might have undergone intense selection pressure during domestication

based on biological status, select geographic regions and countries. ROD values in combinations of *C. reticulatum* accessions versus landraces, landraces versus breeding lines, and breeding lines versus cultivars were quantified using genetic diversity (π). To identify the genomic regions influenced by domestication, breeding and cultivation, ROD values were calculated in a sliding window of 10 kb window with a 2 kb sliding window (10 kb/2 kb windows), as described earlier[2]. ROD values were calculated for *C. reticulatum* accessions (28) versus landraces from the most likely origin of domestication: Turkey (139), Syria (80), Iraq (15) and Iran (549). ROD values were also calculated in combinations of landraces from South Asia (875), the Middle East (314), and the Mediterranean (179) versus breeding lines (396).

In terms of biological status, we identified 2,899 (42,148 kb), 191 (4,360 kb) and 14 (404 kb) regions containing 4,567; 419; and 38 candidate genes for *C. reticulatum* vs landraces, landraces vs breeding lines and breeding lines vs cultivars, respectively (Supplementary Data 6 Table 3). In the cases of Mediterranean, Middle Eastern and South Asian regions, 50 (746 kb), 72 (1,044 kb), and 2 (26 kb) regions underwent selection in landraces vs breeding lines, containing 72, 109, and 2 candidate genes, respectively (Supplementary Data 6 Table 4). In the case of *C. reticulatum* vs landraces from Iran, Iraq, Syria and Turkey, we observed 222 (2,532 kb), 4 (46 kb), 13 (142 kb) and 66 (776 kb) regions containing 207, 3, 9 and 48 genes, respectively (Supplementary Data 6 Table 5).

Genetic loads

Bottlenecks and selection might have led to the fixation of deleterious alleles in chickpea. To understand rare allele burden (i.e., genetic loads) and fitness loss in chickpea, we aligned the chickpea genome using the LASTZ[77] tool v1.4.00 with those of Medicago[18], Arabidopsis[70], pigeonpea[69], soybean[68], lotus[78] (*Lotus japonicus*), and common bean[79] (*Phaseolus vulgaris*) to identify genomic regions constrained during evolution. Multiple whole genome sequence alignments were used to calculate rejected substitutions (RS) scores using the GERP++ (May 22, 2011) program[80]. For determining the intensity of constrained regions at each base position, we used a phylogenetic tree and 4DTv (four-fold degenerate transversion) analysis as described earlier[8]. Genomic evolutionary rate profiling (GERP) analysis identified 29 Mb of the analyzed 347 Mb chickpea genome (8.36%) as evolutionarily constrained (GERP score >0) (Extended Data Fig. 8a). Chickpea's evolutionarily constrained genome portion is smaller than cassava[8] (~100 Mb, 20% of the genome) but larger than maize[81] (~105 Mb, 5.42% of the genome).

We used genomic evolution and amino acid conservation modelling in the identified constrained genome to predict putative deleterious mutations in chickpea. We report 58,794 synonymous and 75,955 non-synonymous SNPs based on SIFT annotations (Extended Data Fig. 8b). By considering SIFT scores <0.05, 25,268 (33.27%), non-synonymous SNPs were identified as putatively deleterious mutations. As the strength of functional prediction methods varies[7], we combined SIFT (<0.05) and GERP (>2) to obtain a more conservative set of 10,616 candidate deleterious mutations in the coding regions of 5,728 chickpea genes.

To estimate the individual mutation burden, we used Medicago, which diverged from the chickpea lineage 10-20 MYA, as an outgroup to identify derived deleterious alleles in chickpea. The derived allele frequency (DAF) spectrum showed that chickpea had fewer fixed deleterious mutations (37, non-synonymous deleterious; SIFT < 0.05; GERP > 2; DAF > 0.8) than cassava[8] (150) (Supplementary Data 8 Table 1). The 37 fixed deleterious alleles were present in 36 genes (Supplementary Data 8 Table 2). These deleterious alleles might not have been purged through traditional breeding, which relies on the recombination of segregating alleles. To increase the fitness of cultivated chickpea, these alleles are potential targets for both genomics-assisted breeding or genome editing. With the other 10,579 segregating deleterious mutations predicted, the mutation burden (10,616) in chickpea is substantial. The higher proportion of constrained genome and deleterious alleles in chickpea than maize but lower than cassava might be explained by different modes of reproduction and their consequences on recombination[82]. Recombination is highest for cross-fertilizing crops like maize, less frequent in self-fertilizing crops like chickpea, and almost absent or limited in asexual (vegetatively propagated) crop species like cassava.

Domestication and breeding play an important role in purging deleterious alleles and reducing the genetic loads. Therefore, the occurrence of 10,616 deleterious mutations was analysed across biological status groups. Our analysis showed 17.88% more deleterious alleles in the wild progenitor species (*C. reticulatum*) than cultivated chickpea accessions (Extended Data Fig. 8c). Similarly, landraces had more deleterious alleles than breeding lines (5.91%) and cultivars (20.27%); and desi accessions showed a greater number of deleterious alleles than kabuli accessions (14.74%), Supplementary Data 8 Table 3).

To understand whether the mutation burden is associated with domestication and breeding bottlenecks, we analysed 18 selective sweeps under domestication, as identified in SweeD[61] (v3.3.3) analysis (Supplementary Data 6 Table 8 and 9), and 6,012 kb selective sweeps under

breeding, identified from ROD, F_{ST} and Tajima's D (Supplementary Data 6 Table 3), for deleterious alleles. The chickpea progenitor species (*C. reticulatum*) had 7.05 times more deleterious alleles than cultivated accessions (Supplementary Data 8 Table 4). Similarly, landraces harboured 3.07 times more deleterious alleles than breeding lines (Extended Data Fig. 8d). These analyses indicate that haplotypes containing fewer deleterious mutations have been preferentially selected during domestication and breeding.

Genome wide association studies

A set of 2,980 cultivated chickpea genotypes along with six checks (four desi genotypes- Annigeri, G 130, ICCV 10 and JG 11; two kabuli genotypes- KAK2 and L550) were phenotyped using augmented block design in six locations, namely Patancheru (17.5287° N, 78.2667° E), Amlaha (23.1208° N, 76.9038° E), Junagadh (21.5222° N, 70.4579° E), Durgapura (23.5204° N, 87.3119° E), Kanpur (26.4499° N, 80.3319° E), and Sehore (23.2032° N, 77.0844° E) in India for two years (2014-15 and 2015-16). A total of 16 agronomic traits [plant height (PLHT, cm), plant stand (PLST), plant width (PLWD, cm), basal primary branch (BPB), apical primary branch (APB), basal secondary branch (BSB), apical secondary branch (ASB), tertiary branch (TB), days to 50% flowering (DF), days to maturity (DM), flowering duration (FD), pods per plant (PPP), seed per pod (SPD), 100 seed weight (100SW, g), yield per plant (YPP, g), plot yield (PY, kg ha^{-1}) were evaluated. GWAS using MLM in GAPIT3[83] v20191108 identified 205 SNPs associated with 11 yield and yield-related traits, called marker-trait associations (MTAs) (Supplementary Data 9 Table. 1). The number of MTAs for various traits varied mainly due to the complex nature of the traits targeted. Most agronomic traits are quantitative (governed by several genes with small phenotypic effects) and show extensive genotype × environment interaction. As a result, one may detect more signals in some environments, and in other environments, there is very low/ no signal for a given trait. Manhattan and QQ-plots for GWAS analysis are available at doi:10.6084/m9.figshare.15015309 and doi:10.6084/m9.figshare.15015315, respectively.

By only considering associations with >10% phenotypic variation, 195 MTAs were designated robust MTAs. However, stable (occurring in more than one location) and consistent (occurring in more than one year) MTAs are more useful for crop breeding. By considering these parameters, 27 MTAs for two traits, 100SW and DM, were identified. Of these 27 MTAs, 23 for 100SW and 4 for DM traits were stable, and 28 MTAs (21 for 100SW and 7 for DM) were consistent (Supplementary Data 9 Table 1).

Of the 205 associated SNPs, 152 were present in 79 unique genes with a putative function. For instance, Ca1_1201584 SNP, showing consistent and stable MTA with 100SW, is present in the *Ca_00148* gene that encodes (3S,6E)-nerolidol synthase 1-like. Another SNP (Ca4_13394035) associated with consistent and stable MTAs for 100SW is present in the *Ca_04561* gene that encodes a zinc finger, C3HC4 type (RING finger) protein (Supplementary Data 9 Table 2). A VIGS analysis showed that the C3HC4-type zinc finger protein affected seed pod development in tobacco[84]. Similarly, SNP locus Ca1_1201584, showing consistent and stable association with 100SW, was found in the Ca*_04566* gene that encodes a BRASSINOSTEROID INSENSITIVE 1-associated receptor kinase 1. Brassinosteroids regulate seed size and shape in Arabidopsis[85]. For DM, we identified 40 significant MTAs, including four stable and seven consistent MTAs. One SNP (Ca5_28008272) associated with a consistent and stable DM MTA is the *Ca_13416* gene that encodes an EARLY FLOWERING 4 protein.

Haplotype analysis

Of the 79 genes associated with 11 target traits, one gene had only one SNP. Therefore, the remaining 78 genes were used for haplotype analysis in 3,171 chickpea genotypes. As a result, we identified 350 haplotypes for 67 genes with 2-23 haplotypes per gene (Supplementary Data 9 Table 3 and 4). Further, by using 19.10 million haplo-pheno combinations for 57 genes (with >2 haplotypes each), we identified 24 consistent and stable superior haplotypes (12 for desi and 12 for kabuli) for 20 genes (Supplementary Data 9 Table 5 and Extended Data Fig. 9a). Of these 20 genes, only four (for two traits, 100SW and PY) were shared between desi and kabuli chickpeas. These results further support the specific breeding program requirements for both types of chickpeas. Furthermore, the lines carrying superior haplotypes had significantly higher performance across locations than those without superior haplotypes (Supplementary Data 9 Table 6 and 7), highlighting the role of superior haplotypes for haplotype-based breeding.

To validate our hypothesis on superior haplotypes, we used historical data on 129 chickpeas (88 desi and 31 kabuli) varieties released during different periods (before 1993 (RP1), 1993-2002 (RP2) and after 2002 (RP3)). Only 24% of varieties (9 desi and 22 kabuli) contained the superior haplotype for either one or a combination of traits (Supplementary Data 9 Table 8). None of the desi varieties contained the superior haplotypes for 100SW, PLHT or PPP. However, for DM, the superior haplotypes (early type) occurred in both RP1 and RP2 desi varieties, reflecting that breeding programs have already been introgressing/ selecting this trait.

For kabuli chickpeas, RP3 varieties had the most superior haplotypes for four traits (100SW, DM, PPP, PY), followed by RP2 for three traits (DM, PPP, PY), and RP1 for two traits (PPP, PY). A comparative analysis of 100SW data showed a significant increase in the average performance of lines over the release period (Extended Data Fig. 9b). The phenotyping performance of RP2 and RP3 varieties showed a significant increase in the varieties with superior 100SW haplotypes at 5 out of 6 locations (Extended Data Fig. 9c). This analysis highlights the success of breeding programs to develop improved varieties by accumulating superior haplotypes even without genomic information. This analysis validates our concept of haplotype-based breeding for transferring superior haplotypes (not yet present in varieties) from landraces, highlighting the value of transferring superior haplotypes not yet present in varieties from landraces to elite varieties. Therefore, we have identified 28 lines each in desi and kabuli cultivars that combine two to four superior haplotypes for the targeted traits (Supplementary Data 9 Table 9). Furthermore, based on location-specific phenotypic performance, we have selected 31 location- and trait-specific donor lines for haplotype-based breeding (Supplementary Data 9 Table 10).

Optimal contribution selection (OCS)

Optimal contribution selection[22] (OCS) combined with a mate allocation method that takes into account genetic gain and genetic diversity serve as a guide to a potential future pre-breeding program or "evolving gene bank" (ref.[22,23]). The GRM and GEBVs for the major groups, desi, kabuli, and intermediate types, provides excellent information for pre-breeding chickpeas; the most critical decision is which parents should be crossed to improve the rate of genetic improvement while maintaining genetic diversity in a chickpea pre-breeding program. In Matesel, the GRM is used to estimate both co-ancestry among candidate genotypes and progeny inbreeding (F). The user decides on the number of matings in each group (desi, kabuli or intermediate), any other constraints to mating, and the relative emphasis on the mean index versus co-ancestry in the progeny[24]. Matesel dictates which individuals to select and the actual mating allocations and/or selfings to be made.

Detailed OCS analysis predicted a relatively high rate of overall genetic gain with an increase in the economic index of US $160.92/ha predicted in the next generation. This was achieved with a mean predicted progeny inbreeding of 0.17 in kabuli and 0.03 in desi, which is consistent with the notion that kabuli candidate parents have higher co-ancestry than desi candidates (Supplementary Data 10 Table 2). These results confirm that new genetic diversity should be

introduced into kabuli breeding to avoid erosion of genetic diversity. OCS is based on an economic index and weightings for YPP, 100SW, DF and DM in index favour selection for large seed size and yield in kabuli types (with little selection for earliness) and earliness and yield in desi types (with little selection for seed size). This can now be done with confidence by combining superior haplotypes from desi and kabuli chickpea.

Genomic prediction approaches

We have used different subsets of SNPs from the 3.94 million SNP dataset and 16 trait phenotyping data over 6 locations on 2,980 cultivated genotypes for genomic prediction using three prediction approaches. We provide methodology in brief and some results for each of these approaches as following:

Estimating GEBVs by consideration of the interaction of genomic and environmental covariates

In this approach, genomic prediction models with environment (E), genotype (L), and marker effects were used to estimate Pearson's correlation coefficient between the observed phenotype and predicted GEBVs using three cross-validations (CV0, CV1, and CV2). In summary, the Bayesian Generalized Linear Regression (BGLR) R-package[64] can include pedigree data in parametric and semiparametric contexts and allows different random matrices with user defined covariance matrices[86] was used in our study. We describe the most complex model used in the analysis (E+L+G+GE) here. The remaining models are particular cases of this model, and these can be easily obtained by omitting some of the terms.

Main effects of environments, lines, and markers and genomic × environment interaction: The response of the phenotypes (y_{ij}) defined by

$$y_{ij} = \mu + E_i + L_j + g_j + gE_{ij} + e_{ij} \qquad (1)$$

where μ is the overall mean, E_i is the random effect of the ith environment, L_j is the random effect of the jth line, g_j is a random variable of the random vector $g = (g_1, \dots, g_J)'$ that has the genomic value of the lines, gE_{ij} is a random interaction effect between the ith environment (E_i) and the jth genomic component (g_j) of the lines, and e_{ij} is the random error term. The random effects E_i, L_j and e_{ij} follow independent and identically distributed (iid) normal densities such

that $E_i \sim N(0, \sigma_E^2)$, $L_j \sim N(0, \sigma_L^2)$, and $e_{ij} \sim N(0, \sigma_e^2)$ where σ_E^2,σ_L^2, $\sigma_e^2$are the environment, line, and residual variances, respectively. For the remaining random effects, we have that $g_j$is a random variable of the random vector $g = (g_1, \ldots, g_J)'$ that has the genomic value of the lines and it is assumed to follow a multivariate normal distribution such that $\boldsymbol{g} = \{g_j\} \sim N(0, G\sigma_g^2)$, where $\sigma_g^2$is the genetic variance of the lines and $G = \frac{XX'}{p}$, with X is the centered and standardized matrix of molecular markers where p represents the number of markers[87]; gE_{ij} represents the interaction between each genomic marker with each environment[21] with $\boldsymbol{gE} = \{gE_{ij}\} \sim N(0, (Z_g G Z_g')^{\circ}(Z_E Z_E')\sigma_{gE}^2)$, Z_g and Z_E are the corresponding incidence matrices for connecting phenotypes with genotypes and environments, respectively; σ_g^2 and σ_{gE}^2 are the corresponding variance components.

As previously indicated, the genomic matrix $\boldsymbol{G}$ is used to account for the genomic main effects and the genotype × environment interaction effect, which could be either derived from SNP markers G1, G2, or G3. Models E+L and E+L+G can be derived from (1) by omitting g_j and $(g_j + gE_{ij})$, respectively.

By considering above mentioned model, we estimated prediction accuracies for different traits (Supplementary Data 10 Table 4). A varied pattern of prediction accuracy for different traits was observed with different models. For 100 SW, the results have been presented in Fig. 3b.

Prediction of trait performance by implementing genotype and geographical coordinates using the WhoGEM

A new approach WhoGEM was implemented as described by Gentzbittel et al.[26] for trait performance by implementing genotype and geographical coordinates. In brief, the WhoGEM approach utilizes information about genotype and geographical coordinates to predict the genotype's phenotypic performance for a location. WhoGEM mainly calculates the relationship between geo- and admixture-based genetic distance to estimate the range of K values of interest and compare each accession's predicted vs reported location.

We used a general linear model to predict the phenotypes using the WhoGEM prediction machine in the reference dataset of cultivated chickpeas to explore the relationships between the phenotypes and the admixture components, land types and environment, with or without interactions. Environments are considered as the combination of Year and Location. A

forward-backwards algorithm was used to reduce the set of predictors to the most significant ones. The model was fitted on the whole dataset, and the significant factors identified and conserved.

$$Phenotype = \mu + \alpha_k \sum_{k=1}^{23} \text{Prop. of Comp. } K_k + \beta_i \text{ LandType}_i + \eta_j \text{Envir}_j + \gamma_{kj} \text{Envir}_j \times \sum_{k=1}^{23} \text{Prop. of Comp. } K_k + \epsilon$$

The model without Admixture Component x Environment interaction, called an additive model or WhoGEM w/o GxE model, is:

$$Phenotype = \mu + \alpha_k \sum_{1}^{23} \text{Prop. of Comp. } K_k + \beta_i \text{ LandType}_i + \eta_j \text{Envir}_j + \epsilon$$

As “negative control”, a model w/o any genetics (called environment-only) is also fitted to the data, namely:

$$Phenotype = \mu + \eta_j \text{Envir}_j + \epsilon$$

In summary, the ADMIXTURE and DAPC methods identified K=23 as the most likely number of admixture components among the 1,318 accessions studied. Clustering the 1,318 accessions, using UPGMA and a Gower distance - a distance that accounts for both genetic diversity estimated from admixture component proportions and seed type, showed that seed type is a major determinant of population structure. We have provided prediction accuracies for different traits in Supplementary Data 10 Table 5 and Extended Data Fig. 11.

Haplotype based local genomic estimated breeding values (local GEBVs)

In this approach, we estimated local GEBVs using haplotypes. We selected 124,833 SNPs for constructing LD blocks/ haplotypes and used them with phenotyping data with an algorithm implemented in the R package *SelectionTools* v19.4 (http://population-genetics.uni-giessen.de/~software/). We calculated pairwise r^2 values between SNP markers across each chromosome and selected the adjacent marker pair with the highest LD among all pairs. If the r^2 of a pair exceeded the threshold of 0.5, then markers were defined as a new LD block. Flanking markers on each side of the LD block were then analyzed and added to the same block

if their pairwise r^2 values with the respective outer markers in the LD block also exceeded the threshold. A tolerance parameter per block of $t = 3$ was set to account for incorrectly positioned markers or biased LD estimates, meaning that if up to three consecutive flanking markers did not fulfil the LD threshold, then the block was extended. If more than $t = 3$ flanking markers had a lower LD than $r^2 = 0.5$, the block was completed. This procedure was repeated until all markers were assigned to blocks. SNPs that were not in LD with any other marker were assigned to individual LD blocks.

We grouped the 3,366 chickpea accessions based on their passport data into three groups 'cultivars' (CV, N=152), 'breeding lines' (BL, N=396), and 'landraces' (LR, N=2,439). Accessions that were not assigned to any group were not considered in the analyses (N=379). Based on these groupings, we created three subgroups s1 (CV), s2 (CV+BL) and s3 (CV+BL+LR). To compare the estimated potential of trait improvement when stacking the best haplotypes across the entire genome, we generated *in silico* genotypes for each trait–trial combination in each subgroup, i.e. 11 'ideal' genotypes for each subgroup for each of the seven traits. We then compared these *in silico* genotypes to the respective accession with the highest GEBV for the trait in the respective field trial and determined the potential genetic gain when expanding the CV subgroup to CV+BL or CV+BL+LR.

Using the above approach, we have presented substantial genetic potential in each subgroup for trait improvement in Extended Data Fig. 12.

References:

67. Qanbari, S. et al. The pattern of linkage disequilibrium in German Holstein cattle. *Anim. Genet.* **41**, 346-356 (2010).
68. Schmutz, J. et al. Genome sequence of the palaeopolyploid soybean. *Nature* **463**, 178-183 (2010).
69. Varshney, R. K. et al. Draft genome sequence of pigeonpea (*Cajanus cajan*), an orphan legume crop of resource-poor farmers. *Nat. Biotechnol.* **30**, 83-89 (2012).
70. Arabidopsis Genome Initiative. Analysis of the genome sequence of the flowering plant *Arabidopsis thaliana*. *Nature* **408**, 796-815 (2000).
71. UniProt Consortium T. UniProt: the universal protein knowledgebase. *Nucleic Acids Res.* **46**, 2699 (2018).
72. Stanke, M., Steinkamp, R., Waack, S. & Morgenstern, B. AUGUSTUS: a web server for gene finding in eukaryotes. *Nucleic Acids Res*. **32**, W309–W312 (2004).
73. Haas, B.J. et al. Automated eukaryotic gene structure annotation using EVidenceModeler and the Program to Assemble Spliced Alignments. *Genome Biol.* **9,** R7 (2008).
74. Li, H. A statistical framework for SNP calling, mutation discovery, association mapping and population genetical parameter estimation from sequencing data. *Bioinformatics* **27,** 2987-2993 (2011).
75. Kale, S. M. et al. Prioritization of candidate genes in *"QTL-hotspot"* region for drought tolerance in chickpea (*Cicer arietinum* L.). *Sci. Rep.* **5**, 15296 (2015).
76. Shan, F., Clarke, H. C., Plummer, J. A., Yan, G. & Siddique, K. H. M. Geographical patterns of genetic variation in the world collection of wild annual *Cicer* characterized by amplified fragment length polymorphisms. *Theor. Appl. Genet.* **110**, 381-391 (2005).
77. Harris, R. S. *Improved pairwise alignment of genomic DNA*. PhD Thesis, Pennsylvania State Univ. (2007).
78. Sato, S. et al. Genome structure of the legume, *Lotus japonicus*. *DNA Res.* **15**, 227-239 (2008).
79. Schmutz, J. et al. A reference genome for common bean and genome-wide analysis of dual domestications. *Nat. Genet.* **46**, 707-713 (2014).
80. Davydov, E. V. et al. Identifying a high fraction of the human genome to be under selective constraint using GERP. *PLoS Comput. Biol.* **6**, e1001025 (2010)

81. Rodgers-Melnick, E. et al. Recombination in diverse maize is stable, predictable, and associated with genetic load. *Proc. Natl Acad. Sci. USA* **112**, 3823-3828 (2015).
82. Charlesworth, B., Charlesworth, D. & Morgan, M. T. Genetic loads and estimates of mutation rates in highly inbred plant populations. *Nature* **347**, 380-382 (1990).
83. Wang, J. & Zhang, Z. GAPIT Version 3: Boosting Power and Accuracy for Genomic Association and Prediction. *bioRxiv* 2020.11.29.403170 (2020)
84. Wu, W., Cheng, Z., Liu, M., Yang, X. & Qiu, D. C3HC4-type RING finger protein Nb ZFP1 is involved in growth and fruit development in *Nicotiana benthamiana*. *PLoS ONE* **9**, e99352 (2014).
85. Jiang, W. B. et al. Brassinosteroid regulates seed size and shape in Arabidopsis. *Plant Physiol.* **162**, 1965-1977 (2013).
86. Pérez -Rodríguez,P.etal. A pedigree-based reaction norm model for prediction of cotton yield in multi-environment trials. *Crop Sci.* **55**, 1143-1151 (2015).
87. Van Raden, P. M. Genomic measures of relationship and inbreeding. *Interbull Annu. Meet. Proc.* **37**, 33–36 (2007).
