# Supplementary material for: A chickpea genetic variation map based on the sequencing of 3,366 genomes
Source: Nature. 2021 Nov 10;599(7886):622–7. doi: 10.1038/s41586-021-04066-1 (PMC8612933; doi:10.1038/s41586-021-04066-1)
Supplement: Supplementary file 2 — Reporting Summary [file 41586_2021_4066_MOESM2_ESM.pdf]

nature portfolio

Corresponding author(s): Rajeev K. Varshney, Xin Liu

Last updated by author(s): Sep 13, 2021

Reporting Summary

Nature Portfolio wishes to improve the reproducibility of the work that we publish. This form provides structure for consistency and transparency in reporting. For further information on Nature Portfolio policies, see our Editorial Policies and the Editorial Policy Checklist.

Statistics

For all statistical analyses, confirm that the following items are present in the figure legend, table legend, main text, or Methods section.

n/a	Confirmed	
☐	☒	The exact sample size (*n*) for each experimental group/condition, given as a discrete number and unit of measurement
☐	☒	A statement on whether measurements were taken from distinct samples or whether the same sample was measured repeatedly
☐	☒	The statistical test(s) used AND whether they are one- or two-sided<br>*Only common tests should be described solely by name; describe more complex techniques in the Methods section.*
☐	☒	A description of all covariates tested
☐	☒	A description of any assumptions or corrections, such as tests of normality and adjustment for multiple comparisons
☐	☒	A full description of the statistical parameters including central tendency (e.g. means) or other basic estimates (e.g. regression coefficient) AND variation (e.g. standard deviation) or associated estimates of uncertainty (e.g. confidence intervals)
☐	☒	For null hypothesis testing, the test statistic (e.g. *F*, *t*, *r*) with confidence intervals, effect sizes, degrees of freedom and *P* value noted<br>*Give P values as exact values whenever suitable.*
☒	☐	For Bayesian analysis, information on the choice of priors and Markov chain Monte Carlo settings
☒	☐	For hierarchical and complex designs, identification of the appropriate level for tests and full reporting of outcomes
☒	☐	Estimates of effect sizes (e.g. Cohen's *d*, Pearson's *r*), indicating how they were calculated

Our web collection on statistics for biologists contains articles on many of the points above.

Software and code

Policy information about availability of computer code

Data collection	Sequencing data was generated on Illumina HiSeq2500.
Data analysis	Admixture (v1.3.0), AUGUSTUS (v3.1), BCFTools (v1.4), BGLR (V1.0.7), BLAST (v2.2.31), BreakDancer (v1.1.2), BUSCO (odb10), BWA (v0.7.15), CD-HIT (v4.81), Control-FREEC (v11.0), EIGENSOFT (v7.2.0), EnrichmentPipeline (https://sourceforge.net/projects/enrichmentpipeline), EVM (v1.1.1), Flapjack (v1.19.09.04), GAPIT3 (v20191108), GATK (v3.7), GATK (v3.8.1), Gblocks (v0.91b), gccount (http://bioinfo-out.curie.fr/projects/freec/src/gccount.tar.gz), GeneWise (v2.4.1), GERP++ (May 22 2011), KEGG (v87.0), LASTZ (v1.4.00), Matesel (v6.3), MCMCTree (v4.4), Megahit (v1.2.9), MUSCLE (v3.8.31), NUCmer (v4.0.0beta2), PHYLIP (v3.6), Pindel (v0.2.5b9), PLINK (v1.90), PopLDdecay (v3.29), RAxML (v8.2.12), rrBLUP (v4.6.0), SAMTools (v1.2), SelectionTools (v19.4), SIFT 4G (v2.0.0), SMC++ (v1.13.1), SweeD (v3.3.1), SWISS-PROT (release-2018_07), VCFtools (v0.1.13).

For manuscripts utilizing custom algorithms or software that are central to the research but not yet described in published literature, software must be made available to editors and reviewers. We strongly encourage code deposition in a community repository (e.g. GitHub). See the Nature Portfolio guidelines for submitting code & software for further information.

Data

Policy information about availability of data

All manuscripts must include a data availability statement. This statement should provide the following information, where applicable:

- Accession codes, unique identifiers, or web links for publicly available datasets
- A description of any restrictions on data availability
- For clinical datasets or third party data, please ensure that the statement adheres to our policy

The data that support the findings of this study has been deposited in the NCBI under accession code BioProject: PRJNA657888. The chickpea pan-genome assembly

nature portfolio | reporting summary

March 2021

and annotations developed in this study are available at doi: 10.6084/m9.figshare.16592819. The variant calls for each accession and phenotype data are available to download at https://cegresources.icrisat.org/cicerseq. Manhattan and QQ-Plots for GWAS analysis are available at doi:10.6084/m9.figshare.15015309 and doi:10.6084/m9.figshare.15015315, respectively. BUSCO (odb10) and SWISS-PROT (release-2018_07).

Field-specific reporting

Please select the one below that is the best fit for your research. If you are not sure, read the appropriate sections before making your selection.

☒ Life sciences ☐ Behavioural & social sciences ☐ Ecological, evolutionary & environmental sciences

For a reference copy of the document with all sections, see nature.com/documents/nr-reporting-summary-flat.pdf

Life sciences study design

All studies must disclose on these points even when the disclosure is negative.

Sample size	We sequenced 3000 accessions from global chickpea composite collection, which was developed by ICRISAT genebank in collaboration with ICARDA to define the genetic structure and represent the maximum diversity for the isolation of allelic variants of candidate gene associated with beneficial traits. The composite collection is a useful resource for detecting new sources of genetic variation and allelic variants of candidate gene(s) associated with beneficial traits, identifying diverse lines for use in functional and comparative genomics, in mapping and cloning gene(s), and in applied breeding (Upadhyaya et al. 2005, Plant Genetic Resources).
Data exclusions	Genotyping data was filtered using various well established criteria including % of missing, minor allele frequency and others. Similarly low quality phenotyping data from 3 site/year combination was filtered out. These exclusion have been defined for each analysis in the Methods section.
Replication	The composite collection, along with very promising checks (Annigeri, G130, ICCV10, JG11, KAK2 & L550) lines, were evaluated in an augmented block design. The experiment was conducted at Six locations Patancheru, Amlaha, Junagadh, Kanpur, Durgapura and Sehore during the post-rainy season of 2014-15 and 2015-16 years. For sequencing data, no replication was attempted.
Randomization	Analysis of the phenotyping data was performed by considering block as random and entry as fixed effects using the restricted maximum likelihood estimation procedure. Different populations in the analysis were defined based on passport information for germplasm accessions. For instance, based on seed type all cultivated (3171) accessions were divided into three populations/groups namely desi, kabuli and intermediate. Similarly, we also grouped accessions based on biological status (wild, landraces, breeding lines and cultivars) and their country of origin.
Blinding	No blinding. All data were processed equally.

Reporting for specific materials, systems and methods

We require information from authors about some types of materials, experimental systems and methods used in many studies. Here, indicate whether each material, system or method listed is relevant to your study. If you are not sure if a list item applies to your research, read the appropriate section before selecting a response.

Materials & experimental systems

n/a	Involved in the study	
☒	☐	Antibodies
☒	☐	Eukaryotic cell lines
☒	☐	Palaeontology and archaeology
☒	☐	Animals and other organisms
☒	☐	Human research participants
☒	☐	Clinical data
☒	☐	Dual use research of concern

Methods

n/a	Involved in the study	
☒	☐	ChIP-seq
☒	☐	Flow cytometry
☒	☐	MRI-based neuroimaging
